# Perspectives on miRNAs as Epigenetic Markers in Osteoporosis and Bone Fracture Risk: A Step Forward in Personalized Diagnosis

**DOI:** 10.3389/fgene.2019.01044

**Published:** 2019-10-30

**Authors:** Michela Bottani, Giuseppe Banfi, Giovanni Lombardi

**Affiliations:** ^1^IRCCS Istituto Ortopedico Galeazzi, Laboratory of Experimental Biochemistry & Moelcular Biology, Milano, Italy; ^2^Vita-Salute San Raffaele University, Milano, Italy; ^3^Department of Physiology & Pharmacology, Gdańsk University of Physical Education & Sport, Gdańsk, Poland

**Keywords:** biomarkers, circulating miRNAs, miRNA signature, extra-analytical variability, sensitivity and specificity, osteopenia/osteoporosis, fracture risk

## Abstract

Aging is associated with an increased incidence of age-related bone diseases. Current diagnostics (e.g., conventional radiology, biochemical markers), because limited in specificity and sensitivity, can distinguish between healthy or osteoporotic subjects but they are unable to discriminate among different underlying causes that lead to the same bone pathological condition (e.g., bone fracture risk). Among recent, more sensitive biomarkers, miRNAs — the non-coding RNAs involved in the epigenetic regulation of gene expression, have emerged as fundamental post-transcriptional modulators of bone development and homeostasis. Each identified miRNA carries out a specific role in osteoblast and osteoclast differentiation and functional pathways (osteomiRs). miRNAs bound to proteins or encapsulated in exosomes and/or microvesicles are released into the bloodstream and biological fluids where they can be detected and measured by highly sensitive and specific methods (e.g., quantitative PCR, next-generation sequencing). As such, miRNAs provide a prompt and easily accessible tool to determine the subject-specific epigenetic environment of a specific condition. Their use as biomarkers opens new frontiers in personalized medicine. While miRNAs circulating levels are lower than those found in the tissue/cell source, their quantification in biological fluids may be strategic in the diagnosis of diseases that affect tissues, such as bone, in which biopsy may be especially challenging. For a biomarker to be valuable in clinical practice and support medical decisions, it must be (easily) measurable, validated by independent studies, and strongly and significantly associated with a disease outcome. Currently, miRNAs analysis does not completely satisfy these criteria, however. Starting from *in vitro* and *in vivo* observations describing their biological role in bone cell development and metabolism, this review describes the potential use of bone-associated circulating miRNAs as biomarkers for determining predisposition, onset, and development of osteoporosis and bone fracture risk. Moreover, the review focuses on their clinical relevance and discusses the pre-analytical, analytical, and post-analytical issues in their measurement, which still limits their routine application. Taken together, research and clinical findings may be helpful for creating miRNA-based diagnostic tools in the diagnosis and treatment of bone diseases.

## Introduction

### Biogenesis of miRNAs and Their Biological Role

MicroRNAs (miRNAs) are short, single-stranded non-coding RNAs (18–22 nucleotides in length) that inhibit gene expression. Lee et al. (1993) discovered in *Caenorhabditis elegans* — a short, single-stranded non-coding RNA (lin-4) that downregulated lin-14 gene expression through a direct antisense RNA–RNA interaction. Since then, miRNAs have been discovered in all living kingdoms (Lagos-Quintana et al., 2001; Reinhart et al., 2002; Cerutti and Casas-Mollano, 2006; Dang et al., 2011; Bloch et al., 2017) and in viruses, as well (Grundhoff and Sullivan, 2011). Among the databases that record the ever growing number of miRNAs being discovered, miRBase (www.mirbase.org) is a comprehensive and constantly updated miRNAs database that provides universal nomenclature, information about sequence, predicted target genes, and additional annotations (Griffiths-Jones et al., 2006). Currently, it contains 38,589 entries, more than 1,900 of which are human.

Though widely discussed, miRNAs biogenesis is not yet fully understood. Briefly, miRNAs are transcribed by RNA polymerase II (Pol II) from encoding sequences (miRNA genes) located within non-coding DNA sequences, introns or untranslated regions (UTR) of protein-coding genes (Ha and Kim, 2014; Hammond, 2015). miRNA genes can be found in clusters within a chromosomal locus; they are transcribed as polycistronic primary transcripts and subsequently processed as single miRNA precursors. miRNAs within the same cluster are thought to target related mRNAs (Lee et al., 2002; Wang et al., 2016). Furthermore, the same miRNA encoding genes can be duplicated in different loci: the derived mature miRNAs (grouped within a miRNA family) have an identical seed region and share the same mRNA targets (Bartel, 2009). A long primary transcript (pri-miRNA) is processed in the nucleus by the RNase III DROSHA-DGCR8 cofactor complex that removes the stem loop-flanking structure generating the ∼60 nt hairpin pre-miRNA.

After its exportation into the cytosol in a process mediated by exportin 5 (EXP5), RNase III DICER cleaves the loop to generate a double stranded (ds) miRNA. One miRNA strand, the passenger strand, is incorporated into the RNA-induced silencing complex (RISC) as a mature miRNA, while the other, the star strand, is degraded. Both strands in some miRNAs are bioactive and each strand is loaded into a RISC. The RISC protein argonaute-2 (AGO-2) is responsible for targeting a specific mRNA based on the complementarity of a 7-nt miRNA sequence (“seed region,” position 2-to-7). The ds miRNA–mRNA complex induces degradation of the target mRNA, inhibition of its translation, and consequent modulation of the downstream cellular processes. Other DICER- or DROSHA-independent non-canonical miRNA biogenesis pathways exist (Ha and Kim, 2014; Hammond, 2015). Finally, miRNAs expression undergoes multilevel regulation: epigenetically in DNA methylation and histone modifications (e.g., histone acetylation) (Saito et al., 2006; Scott et al., 2006; Lujambio et al., 2008; Lujambio and Esteller, 2009) and through the regulation of proteins involved in miRNAs maturation (Davis-Dusenbery and Hata, 2010). Beside their more known inhibitory function, there are evidence suggesting that at least some miRNAs can induce gene expression under specific conditions. In this process, miRNA-associated ribonucleoproteins (miRNPs) play a key role as reviewed in (Valinezhad Orang et al., 2014).

One of the first demonstrations of the key role of miRNAs was the embryonic lethality of the DICER-1- and DGCR8-double knockout (KO) in mice (Bernstein et al., 2003; Wang et al., 2007). Conditional inactivation of DICER in mice embryonic stem (ES) impaired proliferation and differentiation and compromised miRNA biogenesis (Suh et al., 2004; Murchison et al., 2005). Several miRNAs display a cell- or tissue-specific expression profile, while others are more widely expressed (Ludwig et al., 2016). Since they are also present in human biological fluids (Weber et al., 2010), their abundance and stability in human serum and plasma prompted the idea for their potential use as biomarkers (Chen et al., 2008).

**Figure 1:**
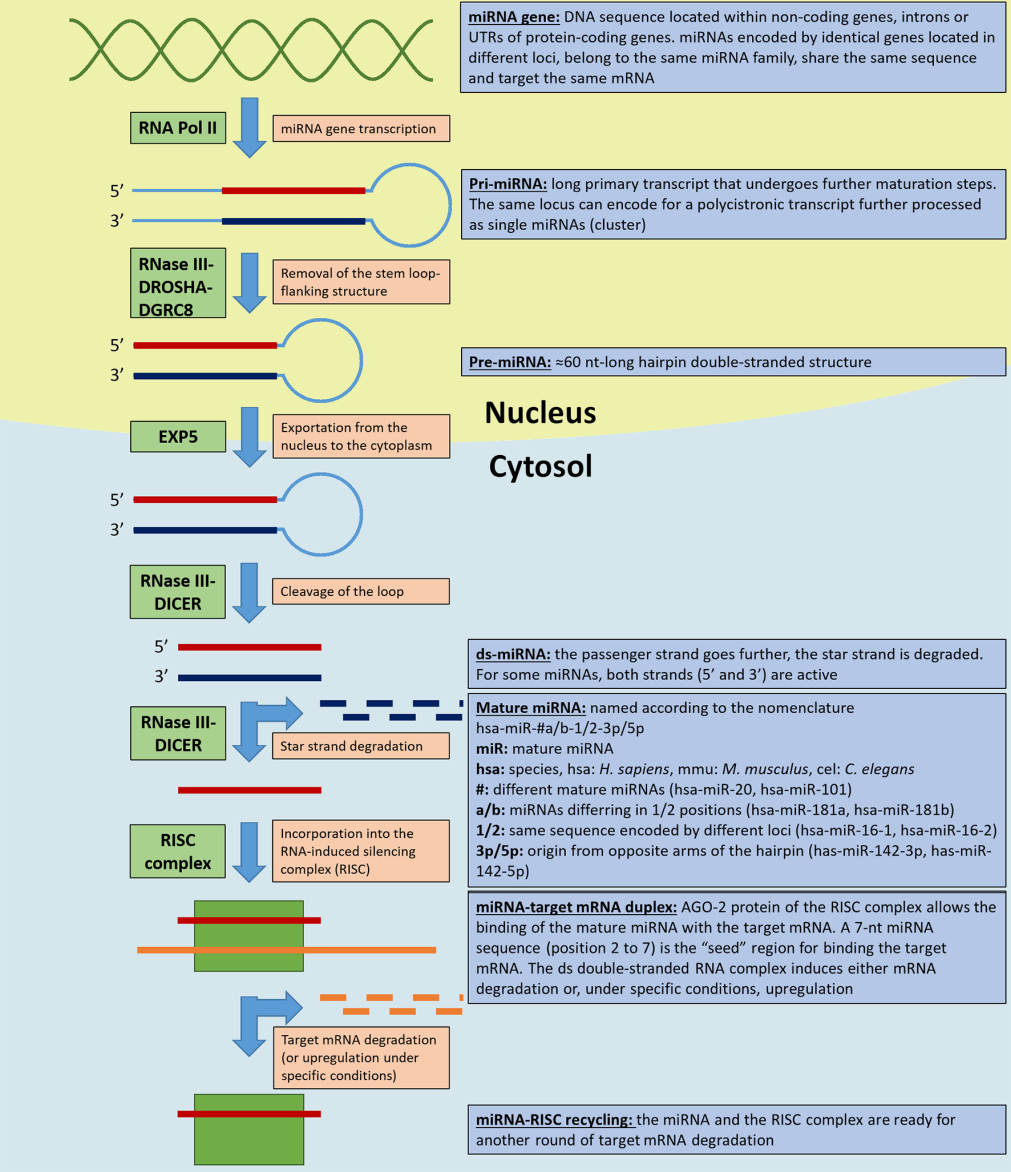
miRNA biogenesis and nomenclature. The figure illustrates the key steps in miRNAs biogenetic canonical pathways. The light orange boxes indicate the step, the green boxes the key enzyme/enzyme complexes involved in the process, and the light blue boxes the miRNAs and miRNAs precursor nomenclature and specifications (according to Griffiths-Jones et al., 2006). RNA Pol II, RNA polymerase II; EXP5, exportin 5; RISC, RNA-induced silencing complex; AGO-2, argonaute-2 protein.

**Figure 1** illustrates the canonical miRNA biogenetic pathway and notions about their nomenclature.

### Aim

Based on the potentialities of miRNAs as biomarkers, research efforts have been spent in studying and defining the relationships between their altered expression and human disease, particularly bone diseases (Bellavia et al., 2019; Hadjiargyrou and Komatsu, 2019; Van Meurs et al., 2019). The search term “miRNA” on PubMed retrieves 83,067 records, 53,240 (64%) of which were published in the last 5 years.

Different from previous reviews, the aim of this paper is to comprehensively review the available data about the potential next use, or even the actual use, of circulating miRNAs as biological indexes for osteoporosis and bone fracture risk. We gleaned information from each article that claimed miRNAs diagnostic, prognostic, and/or predictive properties, including information about the pre-analytical phase, quantification platforms, and normalization methods used. Several articles also reported the sensitivity and specificity parameters in evaluating the clinical potential of a specific miRNA as a biomarker to assess the presence of disease and, at the same time, the absence of the disease in healthy individuals. Since sensitivity and specificity are inversely correlated, they can be plotted on a receiver operating characteristic (ROC) curve as 1-specificity vs. sensitivity (Hajian-Tilaki, 2013).

miRNA can be found in human biofluids and in blood as free (mainly protein-associated) and exosome-/microvesicle-/LDL-associated miRNAs. These two distinct subsets are believed to exert different functions: the free fraction is somehow passively released from cells during normal recycling of the subcellular components, whereas the encapsulated fraction is actively released and finely packaged together with other components with specific functions addressed to other target tissues. In these terms, free-miRNAs can be considered classical biomarkers, while encapsulated miRNAs more likely act as endocrine-like factors (Bayraktar et al., 2017). This review will discuss bone tissue and bone-associated free-circulating miRNAs in relation to osteoporosis and the related risk of bone fracture. In addition, the review will systematically describe the *in vivo–in vitro* evidence for the role, the pathways, and the putative target genes of these miRNAs.

## miRNAs as Biomarkers

Borrowing from Morrow and de Lemos (2007), the three essential features of a novel cardiovascular biomarker for clinical use are: measurability in a certain clinical setting; validation by multiple studies; and direct impact on medical decision making and patient management.

The measurability criterion requires an accurate and reproducible analytical method that can provide reliable measures rapidly and at reasonable cost. Furthermore, pre-analytical issues (conditions of measurement and sample handling, type, and stability) must be known and solved beforehand in order to control for variables in the biomarker’s measurability/detectability. The validation criterion requires a strong and consistent association between the outcome/disease of interest and the biomarker level based on evidence from multiple clinical studies. Moreover, in order to directly impact medical decision making, a novel biomarker must perform better than existing tests and the associated risk might be modified by a specific therapy (Morrow and de Lemos, 2007). These criteria are still burdened by several issues regarding the pre-analytical, analytical, and post-analytical phases in miRNAs.

### miRNAs as Biomarkers: Strengths

These limitations notwithstanding, the use of circulating (or also tissue) miRNAs as biomarkers is nearly ready for implementation in clinical practice. Interest in these molecules arises from the fact that, as epigenetic regulators of gene expression, they act as modulators rather than effectors of a specific biological function. As such, they provide a prompt and easily accessible tool to determine the epigenetic environment of a specific condition. And as subject-specific epigenetic determinants of a condition, they can be considered a personalized signature for tailor-made diagnosis and/or treatment. Circulating miRNAs are easily detectable in biofluids such as (but not only) plasma, serum, and urine, which are minimal/non-invasive sources of biomarkers with broad applicability in clinical research and repositories (Weber et al., 2010; Hackl et al., 2016). Although circulating miRNAs levels are lower than those found in tissues and cells (Jarry et al., 2014), this feature is advantageous, especially in diseases affecting tissues such as bone in which biopsy may be problematic (Hackl et al., 2016). Furthermore, circulating miRNAs can be detected with reliable methods based on polymerase chain reaction (PCR); reverse transcription quantitative PCR (RT-qPCR) is the most widely used owing to its high sensitivity, specificity, and reproducibility (Bustin and Nolan, 2004). Another important advantage of miRNAs as biomarkers is their stability in biofluids due to their encapsulation in extracellular vesicles (ectosomes or exosomes) and in high-density lipoproteins (HDL) and their association with proteins (Argonaute2 or nucleophosmin); miRNAs packaging is correlated with the way they are taken up by target cells (Arroyo et al., 2011; Chen et al., 2012; Li et al., 2012). miRNAs concentration in plasma, as evaluated by qPCR, is highly variable. El-Hefnawy et al. (2004) detected miRNAs concentration in the range of 1–10 µg/L, while Weber et al. (2010) reported a median concentration of 308 µg/L. Differences among healthy humans are physiological and any variation in blood processing conditions can affect circulating miRNA levels (Mitchell et al., 2008; Kroh et al., 2010; Cheng et al., 2013a).

### miRNAs as Biomarkers: Weaknesses

#### Pre-Analytical Issues in miRNA Evaluation

In the pre-analytical phase, two sets of variables can affect miRNAs evaluation: patient-related and sampling-related factors.

##### Patient-related factors: lifestyle habits and diseases

Among patient-related factors, lifestyle habits and diseases affect circulating miRNA levels. Studies have shown that cigarette smoking (Takahashi et al., 2013), physical activity (Baggish et al., 2011; Faraldi et al., 2019), diet (Witwer, 2012), vitamin D levels (Bellavia et al., 2016; Bellavia et al., 2019), and head-down tilt (HDT) bed rest (Ling et al., 2017) can modify the level of a specific miRNA in circulation, whereas gender does not seem to significantly contribute to total variability (Chen et al., 2008). Also, miRNA levels are affected by circadian rhythm (Shende et al., 2011).

The total amount of circulating miRNAs is reduced in chronic kidney disease patients (Neal et al., 2011), while its correlation with liver disease is unknown (Hackl et al., 2016). As a consequence, any clinical study validating a panel of circulating miRNAs as biomarkers must follow pre-analytical protocols with strict criteria for sample collection (preferentially in the morning) and for patient inclusion and exclusion (type of diet, glomerular filtration rate, and fasting time before sample collection) to minimize the effect of variables on the validation process (Hackl et al., 2016).

##### Sampling-related factors: source/matrix, sample collection, and handling

A key step in the validation of a novel biomarker is selection of the correct matrix (Livesey et al., 2008; Kavsak and Hammett-Stabler, 2014). Serum and plasma miRNAs evaluated in the same blood sample are stable, and measurements in healthy individuals are reproducible, consistent, and linkable (Chen et al., 2008; Mitchell et al., 2008). In blood sample collection and handling, phlebotomy is the chief source of variability and contamination with non-circulating miRNAs (Kroh et al., 2010; Cheng et al., 2013a). In detail, miRNA quantification can be affected by the type of collection tube and anticoagulant coating, in addition to blood cell count, needle gauge (Kroh et al., 2010), and hemolysis (Kirschner et al., 2011). Since the total amount of miRNAs contained in cells is considerably higher than in circulation, quantification of circulating miRNAs can be affected by the signal coming from non-circulating miRNA contamination (e.g., the skin contaminant within the needle). In addition, miRNAs can be released by activated platelets or by hemolytic erythrocytes (Kirschner et al., 2011; Willeit et al., 2013). Another often unconsidered source of variability is tourniquet application, together with clenching the fist and maintaining it closed, that can alter blood levels of electrolytes, muscle enzymes, free hemoglobin, water, and low-molecular-weight molecules. Also at the needle insert site the concentration of some blood analytes may be increased (Lima-Oliveira et al., 2013; Lima-Oliveira et al., 2016). For the collection of plasma samples, it is important to use the right anticoagulant: heparin, potassium ethylendiaminotetraacetate (K2/K3 EDTA), sodium fluoride/potassium oxalate (NaF/KOx), or sodium citrate. Heparin (Garcia et al., 2002; Boeckel et al., 2013) and sodium citrate are not recommended for RT-qPCR-based miRNA quantification because they alter the activity of the enzymes used in PCR-based assays (Hackl et al., 2016). Conversely, EDTA is considered the right choice for PCR-based miRNA evaluation because it is easily removed from the PCR mastermix (Zampetaki and Mayr, 2012). Alternatively, NaF/KOx may be used when EDTA is not available, although it can increase the miRNA detection rate (Kim et al., 2012). Centrifugation speed and length to separate plasma can affect miRNAs detection in EDTA-plasma possibly due to platelet-derived miRNAs (Cheng et al., 2013a), while miRNAs evaluation in serum samples is less sensitive to this process (Hackl et al., 2016). miRNAs in blood samples are stable up to 24 h at room temperature (Mitchell et al., 2008) due to their association with proteins or extracellular vesicles. This is important in clinical routine, especially when unexpected delays prolong turnaround time. Interestingly, miRNAs are reported to be stable also in extreme conditions (e.g., low and high pH) or after repeated freezing/thawing cycles (Chen et al., 2008). The ongoing discovery of novel miRNAs, together with the limited number of stability tests, calls for the need of standardized protocols in sample collection and handling in order to minimize pre-analytical sources of error (Cheng et al., 2013a). Samples can be stored for decades at low temperatures (i.e., < −70°C), which facilitates the retrieval of reliable data in retrospective studies (Zampetaki and Mayr, 2012).

#### Analytical and Post-Analytical Issues in miRNA Evaluation

In their study comparing 12 commercially available platforms for evaluating miRNA expression levels (7 PCR-based, 3 microarrays, and 2 next generation sequencing [NGS] technologies), Mestdagh et al. (2014) observed marked differences between the platforms. Because different technologies are often used during the validation process, platform choice will affect a method’s reproducibility and specificity. For any platform combination, the average validation rate for deregulated miRNA expression is 54.6%, indicating that screening studies and validation studies on different platforms and/or technologies must be performed. Sensitivity is more technology-correlated, with qPCR platforms showing the best score and, as a consequence, higher accuracy and more reliable results. These observations suggest that analytical protocols and platforms must be the same for the discovery and the validation of a biomarker and that further efforts are required to aid in the migration to a final commercial platform (Hackl et al., 2016).

The major post-analytical issues in miRNAs evaluation are data normalization and choice of the right reference gene. Presently, there is no consensus on either issue. The amount of miRNAs in a biofluid is expressed in relative rather than absolute terms by volume unit. This makes it hard to compare results across different labs or across different studies performed in the same lab (Nelson et al., 2008; Hackl et al., 2016). The most common normalization methods for miRNAs expression of RT-qPCR data (reviewed in Faraldi et al., 2018) are based on: exogenous synthetic oligonucleotides; endogenous reference genes; and the average of all the miRNA expressed. The right choice of normalization strategy is crucial to reduce analytical variability and to obtain reliable and reproducible results. Exogenous reference genes are non-human synthetic oligonucleotides usually added to the analyzed biological sample to monitor the efficiency and quality of RNA processing.

In miRNAs quantification, the normalization strategies adopted for RT-qPCR data calculation are based on the use of a single reference gene (i.e., cel-miR-238, cel-miR-39, cel-miR-54) (Ho et al., 2010; Wang et al., 2015; Yang et al., 2017) or on the average of multiple reference exogenous oligonucleotides (Mitchell et al., 2008; Sourvinou et al., 2013). These normalization methods have an important limitation, however: unlike endogenous miRNAs, exogenous oligonucleotides are not affected by pre-analytical variables, consequently, they reduce the analytical but not the pre-analytical variability. The use of one or more endogenous reference genes satisfies this criterion because the genes are affected by the same pre-analytical variables as the same analytical procedures of the target miRNA(s); therefore, this is the most suitable normalization strategy for miRNAs data from RT-qPCR-based quantification techniques (Faraldi et al., 2018).

In human samples, the most commonly used endogenous reference gene is has-miR-16 (Faraldi et al., 2018), but several studies have shown very variable expression between cases and controls and the effect of hemolysis on its levels in blood samples (Hu et al., 2012; Liu et al., 2012; Kirschner et al., 2013). Also for endogenous sequences, the normalization method based on the use of multiple reference genes, identified with the aid of informatics tools, is thought to reduce post-analytical variability (Vandesompele et al., 2002; Andersen et al., 2004). With this procedure, however, the miRNAs set as reference cannot be used later in the analysis as targets (Faraldi et al., 2018). Finally, for large amounts of data or in the absence of an *a priori* reference gene, a commonly applied strategy is to calculate the average expression of all the evaluated endogenous miRNA (Mestdagh et al., 2009). Based on these considerations, it is of key importance to standardize the normalization method by determining the most stable reference gene(s) in each experimental setting (Faraldi et al., 2018). Recently, we demonstrated large differences in results obtained by applying different normalization strategies to RT-qPCR data from a panel of 179 circulating miRNAs. Based on analysis of the between-assay coefficients of variation (CV) and of the CV distribution frequencies, we defined the normalization of a specific miRNA (hsa-miR-320d) as the best strategy in that specific setting (Faraldi et al., 2019).

Specific guidelines to standardize pre-analytical, analytical, and post-analytical variables are desirable in order to obtain reliable and comparable miRNA expression data and to accelerate the definitive clinical implementation of miRNAs-based tests.

## miRNAs as Biomarkers for Bone Diseases

While the multiple roles exerted by tissue and exome/microvesicle-associated miRNAs in bone pathophysiology have been identified and validated, the clinical usefulness of circulating miRNAs in skeletal and muscle-skeletal diseases has not yet been established. This is because studies so far have been designed with a mechanistic purpose in mind and not for identifying circulating miRNAs with diagnostic/prognostic abilities for bone fracture risk or treatment response (Hackl et al., 2016). The potential role of circulating miRNAs as biomarkers for the early identification of altered bone metabolism ranks high on the clinical research agenda, given the aging population and the growing incidence of age-associated diseases (e.g., metabolic bone diseases and osteoporosis) and the related risk of bone fracture. Reliable diagnostic tools that can prognosticate a subject-specific risk of disease onset or, if already overt, a subject-specific risk of progression and response to therapy are currently lacking. Furthermore, the natural history of age-associated bone diseases is, as never before, tied to a plethora of subject-specific variables. miRNAs and their circulating fraction hold promise: as epigenetic modifiers of gene expression they act much more upstream of the expression process than classical protein markers. This means that changes in their expression, which are likely to be mirrored by changes in their circulating levels, are effective far before their translation into metabolic and structural changes (Materozzi et al., 2018).

### Circulating miRNAs and Postmenopausal Osteoporosis

Osteoporosis (OP), one of the most prevalent bone diseases, is characterized by impaired bone strength and quality that increase the risk of bone fracture (NIH, 2001). Currently, dual energy X-ray absorptiometry (DXA) is the diagnostic gold standard, while bone turnover markers are useful in framing the metabolic activity of bone cells [e.g., C-terminal cross-link (CTx), N-terminal pro-peptide of type I collagen (PINP), parathyroid hormone (PTH), bone alkaline phosphatase (BAP), osteocalcin, and tartrate-resistant acid phosphatase 5b (TRAP5b), pyridonline/deoxypyridinoline] and in evaluating the effectiveness of anti-resorptive therapies (Lombardi et al., 2012; Vasikaran and Chubb, 2016). Although valuable, these diagnostic tools have several practical flaws that partially limit their utility: on the one hand, radiological methods can reveal only already established bony architectural modifications, which take several weeks or months to become detectable, and on the other, bone turnover markers are not fully specific for either bone or the metabolic process they are associated with (i.e., formation or resorption) (Lombardi et al., 2012).

Despite limitations in pre-analytical, analytical, and post-analytical standardization, miRNAs still have enormous potential in this setting. Indeed, based on their role as highly sensitive fine-tuners of biological processes, when assayed in combination with conventional diagnostics, they may give a more detailed clinical framing and a prompt measure of response to therapy (Faraldi et al., 2018; Sansoni et al., 2018). This is particularly desirable in complex syndromic conditions, such as OP, in which the prognosis (i.e., bone fracture) depends not only upon the bony metabolic status but also on the whole-body metabolism. Circulating miRNAs can much better describe such a complex network. The still limited information about the role of miRNAs in OP is derived from different types of human samples [serum, circulating monocytes or bone marrow-derived mesenchymal stem cells (BM-MSCs), and bone tissue] obtained from patients of different ethnic groups with low bone mineral density (BMD) or bone fractures and compared with healthy controls or osteoarthritis (OA) patients. Furthermore, differences in quantification platforms and normalization processes make it very hard to compare the study data.

Early evidence that OP correlates with altered expression of circulating miRNAs stems from a microarray analysis of 365 miRNAs in human circulating monocytes collected from postmenopausal Caucasian women with either low or high BMD. Of the 365 miRNAs screened by RT-qPCR analysis, only miR-133a was found significantly upregulated in the low-BMD subjects compared with their normal BMD counterparts (Wang et al., 2012). Using the same experimental protocol, the same authors found another marginally expressed miRNA associated with low BMD: miR-422a (Cao et al., 2014). Supporting the hypothesis for their tissue-specificity, subsequent analysis of miR-133a and miR-422a expression in isolated circulating B cells derived from the same subjects disclosed no difference between the two groups (Wang et al., 2012; Cao et al., 2014). Based on these results, the authors speculated that these two miRNAs might be monocyte-specific biomarkers for postmenopausal OP. Mature miR-133a is transcribed from two different loci (18q11.2 and 20q13.33). It was previously described as an inhibitor of osteoblast differentiation by directly targeting RUNX2 in murine pre-myogenic C2C12 and pre-osteoblastic MC3T3-E1 cells (Li et al., 2008; Zhang et al., 2011b). The miR-422a expression level in osteoblast-like cells was described to be decreased after treatment with peptide-15, a factor that increases bone development (Palmieri et al., 2008). Since monocytes are osteoclast precursors, a bioinformatics analysis has highlighted three osteoclast-related potential target genes for miR-133a (CXCL11, CXCR3, and SLC39A1) and five for miR-422a (CBL, CD226, IGF1, PAG1, TOB2) (Wang et al., 2012; Cao et al., 2014). The latter studies, however, suffered from several limitations: limited sample size (10 subjects per group); no evidence of a correlation between miR-133a or miR-422a and target gene expression; and no information about the stem-loop arm of origin of these miRNAs.

In another study, Chen et al. (2014a) evaluated the expression profile of 721 human miRNAs in CD14+ mononuclear cells from peripheral blood (PBMCs) collected from postmenopausal OP women. They found seven differentially expressed miRNAs compared with the non-OP group: four (miR-218, miR-503, miR-305, and miR-618) were downregulated and three (miR-107, miR-133a, and miR-411) were upregulated. Also, miR-133a was confirmed as upregulated in circulating monocytes from postmenopausal OP women (Wang et al., 2012); however, only miR-503, the most deregulated one, was validated by RT-qPCR, and its anti-osteoclastogenic effects were investigated *in vivo* and *in vitro*. Overexpression of miR-503, after pre-miR-503 transfection in OP-derived CD14+, drastically inhibited M‐CSF/RANKL-induced osteoclastogenesis, while its suppression by antagomiR-503 promoted osteoclast differentiation. The authors identified and validated RANK mRNA as a target for miR-503. Furthermore, in ovariectomized (OVX) mice, antagomiR-503 increased RANK protein expression, and promoted bone loss and resorption, whereas agomiR-503 prevented bone loss and resorption (Chen et al., 2014a). Because miR-503 downregulation has a key role in postmenopausal OP onset, it may be a target for new therapeutic strategies for OP.

Using a different approach, a study evaluated the miRNA profile differences in human *bone marrow*-derived mesenchymal stromal cells (BM-MCSs) from OP patients and non-OP controls. In this case, 1,040 miRNAs were screened using a microarray in BM-MCSs collected from healthy premenopausal women (control group, n = 5) and postmenopausal OP women (n = 5) (Yang et al., 2013). Following RT-qPCR validation, miR-21 was found downregulated in the OP women, as confirmed in the MSCs from OVX mice. Further experiments revealed that Spry1 negatively regulates fibroblast growth factor (FGF) and extracellular signal-regulated kinase–mitogen-activated protein kinase (ERK-MAPK) signaling pathways and that it is directly targeted by miR-21. As a consequence, the TNFα-mediated inhibition of miR-21 may impair bone formation, as observed in OP induced by estrogen deficiency. This mRNA seems to be a main regulator of osteoblastic differentiation of MSCs and in postmenopausal OP onset (Yang et al., 2013). Moreover, osteoclast precursors express miR-21, which is upregulated during TNF-α/RANKL-induced osteoclastogenesis (Sugatani et al., 2011; Kagiya and Nakamura, 2013). miR-21 expression is upregulated by the osteoclastogenesis transcription factor c-Fos that binds the miR-21 promoter (Kagiya and Nakamura, 2013) which, in turn, downregulates c-Fos inhibitor-programmed cell death 4 (PDCD4). This positive c-Fos/miR-21/PDCD4 feedback loop regulates and promotes RANKL-induced osteoclastogenesis (Sugatani et al., 2011). In addition, miR-21 is involved in estrogen-induced osteoclasts apoptosis: estrogens inhibit miR-21 expression by inducing Fas-ligand (FasL), another miR-21 target, which in turn inhibits osteoclastogenesis and promotes osteoclast apoptosis (Garcia Palacios et al., 2005; Sugatani and Hruska, 2013).

More recent studies have been focused on whole blood, serum or plasma miRNA profiling in patients with or without OP. Circulating levels of miR-133a, miR-146a, and miR-21 have been assayed by RT-qPCR in plasma samples of Chinese postmenopausal women, grouped as normal, osteopenic or OP. miR-21 was downregulated while miR133a was upregulated in the OP and osteopenic women compared with the controls and both correlated with BMD; miR-146a was unchanged (Li et al., 2014). miR-21 was found downregulated in the BM-MCSs of postmenopausal OP women (Yang et al., 2013), while the monocyte expression of miR-133a was associated with low BMD values (Wang et al., 2012). A study investigated the discriminatory potential between OP and osteopenia of six miRNAs (miR-130b-3p, miR-151a-3p, miR-151b, miR-194-5p, and miR-590-5p) which were found upregulated in OP. Of these six, miR-194-5p was the most upregulated and its expression negatively correlated with BMD. The association between miR-194-5p circulating levels and BMD was later confirmed in a wider cohort of Chinese postmenopausal women with normal, osteopenia, and OP ranges of BMD. The study also reported that miR-194-5p may influence the TGF-β and Wnt signaling pathways, thus acting as a critical factor in the pathophysiology of postmenopausal OP (Meng et al., 2015).

The overexpression of miR-194-5p in mice BM-MSCs was correlated with osteogenesis by targeting both COUP-TFII (chicken ovalbumin upstream promoter-transcription factor II) (Jeong et al., 2014) and STAT1 (signal transducer and activator of transcription 1) (Li et al., 2015b). In parallel, among other 851 miRNAs, miR-27a was validated as the most downregulated one in the serum of postmenopausal OP women compared with their healthy counterparts (You et al., 2016). The MSCs collected from these OP patients displayed an increased adipogenic potential at the expense of osteoblast formation. During osteogenesis, miR-27a is upregulated in MSCs, whereas the opposite occurs during adipogenesis; and indeed, miR-27a silencing in mice impairs bone formation. Myocyte enhancer factor 2c (Mef2c), a transcription factor involved in developmental processes, has been identified and validated as a miR-27a target gene (You et al., 2016). Consistent with previous observations (Lin et al., 2009; Wang and Xu, 2010; Pan et al., 2014), miR-27a expression, is essential for osteoblastic differentiation of MSCs and its downregulation *in vivo* has been associated with bone loss. Bedene et al. (2016) identified, among other nine miRNAs, miR-148a-3p as a potential biomarker for postmenopausal OP based on its significantly higher levels in the plasma samples from OP subjects compared with controls. In CD14+ PBMCs, the RANKL-induced osteoclast differentiation promotes miR-148a expression dependent on the repression of V-maf musculoaponeurotic fibrosarcoma oncogene homolog B (MAFB), a transcription factor whose expression inhibits osteoclastogenesis (Cheng et al., 2013b). miR-148-3p has been found upregulated also in CD14+ PBMCs of patients with systemic lupus erythematous (SLE) in which it was correlated with reduced BMD. Furthermore, treatment of OVX mice with antagomiR-148a slowed bone resorption and increased bone mass (Cheng et al., 2013b). The expression levels of the nine miRNAs assayed by Bedene et al. (2016) revealed that plasma miR-126-3p is also positively associated with BMD at the distal forearm and that miR-423-5p plasma levels are negatively correlated with the 10-year probability of bone fracture in OP.

Using a different approach, Chen et al. (2016) screened a wide range of miRNAs in serum samples from OP mice in order to identify the most stable reference gene (miR-25-3p) for use in data normalization in humans. Fifteen of the screened miRNAs found differentially expressed in the OP mice were then investigated in serum samples from postmenopausal women (7 osteopenic, 10 OP, and 19 healthy women). miR-30b-5p was significantly lower in both the osteopenia and OP samples, while miR-103-3p, miR-142-3p, and miR-328-3p were significantly lower in the OP group only compared with the healthy subjects. The role of miR-103-3p and miR-30b-5p in bone physiology has been validated in *in vitro* studies of osteogenesis: miR-30b-5p expression, whose target is Runx2, decreases during late-stage osteoblast differentiation (Eguchi et al., 2013), while miR-103-3p inhibits osteoblasts differentiation and proliferation by directly targeting Runx2 (Zuo et al., 2015) and Cav1.2 (Sun et al., 2015), respectively. Despite the limited sample size, the serum levels of these four miRNAs in OP patients were positively correlated with BMD. The ROC analysis revealed their diagnostic potential for OP based on the following AUC–sensitivity–specificity values: 0.800–80%–72.2% (miR-103-3p), 0.789–70%–79.0% (miR-142-3p), 0.793–70.6%–79.0% (miR-30b-5p), and 0.874–80%–100% (miR-328-3p) (Chen et al., 2016).

In a study series, circulating monocytes from 12 postmenopausal Mexican-Mestizo women, divided in normal (control group) and OP groups were assayed using a microarray platform for the expression profile of 2,578 miRNAs. The results showed that the three most upregulated miRNAs in the OP group were miR-1270, miR-548x-3p, and miR-8084, while the three most downregulated were miR-6124, miR-6165, and miR-6824-5p. Among the upregulated miRNAs, only miR-1270 was further validated. Based on bioinformatics analysis, nine genes have been identified as possible targets of miR-1270, and RT-qPCR finally validated the interferon regulatory factor-8 (IRF8) gene, an inhibitor of osteoclastogenesis (Zhao et al., 2009; Jimenez-Ortega et al., 2017; Saito et al., 2017), which was significantly downregulated in the OP group. The same research team discovered another monocytic miRNA, miR-708-5p, as a potential biomarker for postmenopausal OP. Next generation sequencing (NGS) of the 46 miRNAs found differentially regulated in the two groups revealed that miR-708-5p and miR-3161 were the two most upregulated in the OP group, whereas miR-4422 and miR-939-3p were the two most downregulated. These four miRNAs were then assayed using RT-qPCR, but only miR-708-5p was validated as it was found significantly upregulated in OP patients compared with controls. Bioinformatics analysis of miR-708-5p disclosed ten potential targets involved in osteoclastogenesis, only five of which (AKT1, AKT2, PARP1, FKBP5, and MP2K3) were effectively downregulated in the OP subjects compared with controls (De-La-Cruz-Montoya et al., 2018). The major limitations besides the small sample size in these two studies were the use of different quantification platforms (microarray and NGS) in preliminary screening of differential miRNA expression and the use of two different normalization strategies for RT-qPCR data analysis. These limitations make it difficult to correlate the data. In any case, miRNA-708-5p and miR-1270 may be suitable biomarkers for postmenopausal OP but require an independent validation study with a larger sample using the same protocol for data quantification and analysis.

The last paper published by this research group is the most complete work to date. The potential of miRNAs as biomarkers for OP was evaluated in serum samples (Ramirez-Salazar et al., 2018). The study was divided in two experimental parts: in the discovery stage, 40 postmenopausal Mexican-Mestizo women (grouped into OP subjects and healthy controls) were recruited, while the validation stage comprised Mexican-Mestizo women with OP, osteopenia, and bone fractures, plus healthy postmenopausal Mexican-Mestizo women. In the discovery stage, microarray analysis of 754 serum miRNAs identified seven miRNAs (miR-1227-3p, miR-139-5p, miR-140-3p, miR-17-5p, miR-197-3p, miR-23b-3p, and miR-885-5p) in which the levels were significantly higher in the OP than in the healthy subjects. Only the three most upregulated (miR-140-3p, miR-23b-3p, and miR-885-5p) were used in the validation stage. The study confirmed by RT-qPCR the higher serum levels of miR-140-3p and miR-23b-3p in the groups with osteopenia, OP or bone fracture, and higher levels of miR-885-5p in the osteopenia group than in healthy subjects. ROC analysis for miR-140-3p and miR-23b-3p, in which their ability to discriminate between OP and healthy women was evaluated, demonstrated that the two miRNAs might be good candidates as biomarkers for BMD loss: AUC of 0.84, 0.96, and 0.92 for miR-140-3p in the osteopenia, OP, and bone fracture group, respectively, compared with the healthy controls, and AUC of 0.73, 0.69, and 0.88, respectively, for miR-23b-3p. Furthermore, miR-140-3p and miR-23b-3p were significantly correlated with BMD in each cohort. Target genes databases predicted AKT1, AKT2, AKT3, BMP2, FOXO3, GSK3B, IL6R, PRKACB, RUNX2, and WNT5B as bone-related genes potentially targeted by miR-140-3p and miR-23b-3p. Other potential osteogenic related target genes have been validated *in vitro* and *in vivo*: SMAD3 (Liu et al., 2016) and RUNX2 (Deng et al., 2017) for miR-23b-3p, and BMP2 (Hwang et al., 2014) for miR-140-5p. The study underlined the importance of miR-140-3p and miR-23b-3p as biomarkers of bone loss and risk of fracture, despite the small sample size especially of the control group.

**Table 1** presents information about circulating miRNAs associated with OP.

**Table 1:** miRNAs related to postmenopausal OP.

Study	Study design	Biomarker source	Sample handling	Quantification platform	Evaluated miRNA	Normalization strategy	Validated miRNA biomarker	Potential target gene	AUC-Sensitivity(%)-Specificity(%)	Limits
(Wang et al., 2012)	20 PM Caucasian women (age 57-68 years): 10 with low BMD (hip/spine Z-score < -0.84); 10 with high BMD (hip/spine Z-score > 0.84)	Circulating monocytes	Monocytes separated by density gradients in UNI-SEP tubes (sodium metrizoate 9.6% and polysucrose 5.6% with 1.077 g/ml density), and isolated using a negative isolation kit	Screening: TaqMan Human MicroRNA Array v1.0 Validation: TaqMan RT-qPCR	Screening: 365 miRNAs tested Validation: miR-133a and miR-382	RNU48	↑miR-133a in low vs. high BMD group	CXCR3, CXCL11, and SLC39A1 (identified for miR-133a using miRDB and TargetScan database but not validated)	/	Small sample size; no significant correlation between the expression level of miR-133a and the potential target genes; no information about the stem-loop arm of miRNA origin; no ROC analysis
(Cao et al., 2014)	21 PM Caucasian women (age 57-68 years): 10 with low BMD (hip/spine Z-score < -0.84); 10 with high BMD (hip/spine Z-score > 0.84)	Circulating monocytes	Monocytes separated by density gradients in UNI-SEP tubes (sodium metrizoate 9.6% and polysucrose 5.6% with 1.077 g/ml density), and isolated using a negative isolation kit	Screening: TaqMan Human MicroRNA Array v1.0 Validation: TaqMan RT-qPCR	Screening: 365 miRNAs tested Validation: miR-27b, miR-422a, miR-151, and miR-152	RNU48	↑miR-422a in low vs. high BMD group	CD226, CBL, IGF1, TOB2, and PAG1 (identified for miR-422a using TargetScan database but not validated)	/	Small sample size; no significant correlation between miR-422a and the evaluated target genes; no information about the stem-loop arm of miRNA origin; no ROC analysis
(Chen et al., 2014a)	31 Chinese PM women with OP and 30 healthy women (age 50-59 years).	PBMCs CD14+	Ficoll-Paque separation step and CD14 antibody-coated magnetic cell sorting MicroBeads used for buffy coat PBMCs isolation and CD14^+^ purification, respectively	Screening: MicroRNA microarray by LC Sciences Validation: SYBR Green RT-qPCR	Screening: 721 miRNAs tested Validation: miR-503	snRNU6	↓miR-503 in OP group vs. non-OP group	RANK (validated as miR-503 target gene)	/	Small sample size; no information about the stem-loop arm of miRNA origin; no ROC analysis
(Yang et al., 2013)	5 OP PM women (age 53-63 years) and 5 premenopausal women (age 39-45 years)	BM-MCSs	Percoll density gradient centrifugation methodology obtaining BM-MCSs from the BM	Screening: LC Sciences microarray platform Validation: RT-qPCR	Screening: 1040 miRNAs tested Validation: miR-21	snRNU6	↓miR-21 in PM OP group vs. non-OP group	SPRY1 (identified for miR-21 using Target Scan 6.0 and Pic Tar databases and validated by *in vitro* experiments)	/	Small sample size; no information about the stem-loop arm of miRNA origin; no ROC analysis
(Li et al., 2014)	40 PM Chinese women with normal, 40 with OP, and 40 with osteopenia range BMD (age 46-69 years)	Cell-free plasma	Plasma obtained from fasting blood samples and stored in liquid nitrogen	miRCURY LNA RT-qPCR	miR-21, miR-133a, and miR-146a	miR-16	↓ miR-21 and ↑ miR-133a in OP and osteopenia groups vs. control	/	/	Small sample size; no information about the used anticoagulant; small sample size; arbitrary decision of the reference gene; no evaluation of the target genes; no information about the stem-loop arm of miRNA origin; no ROC analysis
(Meng et al., 2015)	Discovery cohort: 25 PM women with OP and 23 PM Chinese women with osteopenia (age 59-70 years)Validation cohort: 24 PM Chinese women with normal, 32 with OP and 30 with osteopenia range BMD (age 59-70 years)	Whole blood	Blood samples lysed using RBC lysis solution and centrifuged for 10 min at 450g	Discovery: Agilent Human miRNA microarray followed by SYBR Green RT-qPCR Validation: SYBR Green RT-qPCR	Discovery cohort: comprehensive miRNA expression analysis (Microarray); miR-130b-3p, miR-151a-3p, miR-151b, miR-194-5p, miR-590-5p, and miR-660-5p (RT-qPCR) Validation cohort: miR-194-5p	snRNU6	↑ miR-130b-3p, miR-151a-3p, miR-151b, miR-194-5p, and miR-590-5p in OP vs. osteopenia (Discovery cohort)↑ miR-194-5p in OP and osteopenia vs. control (Validation cohort)	/	/	Small sample size; no evaluation of the target genes; no ROC analysis
(You et al., 2016)	155 PM Chinese women with PM OP (n = 81, age 51-62 years) or healthy (n = 74, age 40-46 years)	Cell-free serum and BM-MSCs	/	Screening: Agilent Human miRNA Microarray Validation: TaqMan RT-qPCR	Screening: 851 miRNAs Validation: miR-27a	snRNU6	↓ miR-27a in OP vs. control	Mef2c (predicted for miR-27a using TargetScan and PicTar database and validated by *in vitro* studies)	/	The mean age of OP and healthy women is significantly different; no information about the stem-loop arm of miRNA origin; no ROC analysis
(Bedene et al., 2016)	74 PM women (age 55-65 years): 57 controls and 17 OP based on femoral neck/lumbar spine/total hip T-score ≤–2.5 SD	Cell-free plasma	Blood samples collected in EDTA tubes, centrifuged at 2800 rpm and 4°C for 10 min, then further centrifuged at 9600g and 4°C for 15 min. Plasma samples stored at -80°C	SYBR Green RT-qPCR	miR-7d-5p, miR-7e-5p, miR-30 d-5p, miR-30e-5p, miR-126-3p, miR-148a-3p, miR-199a-3p, miR-423-5p, and miR-574-5p	Combination of let-7a-5p and miR-16-5p as identified by Normfinder	↑ miR-148a-3p in OP vs. control	/	/	Small sample size of the control group; no evaluation of the target genes; no ROC analysis
(Chen et al., 2016)	36 PM women: 19 HC, 7 osteopenic, 10 OP	Cell-free serum	Serum obtained by centrifuging blood samples in two steps: for 10 min at 2000g and 4°C and for 20 min at 12000g and 4°C. Serum stored at -80°C	SYBR RT-qPCR	miR-30a-5p, miR-30e-5p, miR-425-5p, miR-142-3p, miR-191a-3p, miR-215, miR-29b-3p, miR-30b-5p, miR-26a-5p, miR-345-5p, miR-361-5p, miR-185-5p, and miR-103-3p	NormFinder and GeNorm identified miR-25-3p as the most stable reference gene in mice models of OP	↓ miR-30b-5p in both osteopenic and OP vs. HC↓ miR-103-3p, miR-328-3p, and miR-142-3p OP vs. HC	/	0.793 (miR-30b-5p) for both OP and osteopenia vs. HC0.793-70.6-79.0 (miR-30b-5p), 0.800-80-72.2 (miR-103-3p), 0.789-70-79.0 (miR-142-3p) and 0.874-80-100 (miR-328-3p) for OP vs. HC	Different number of subjects recruited in the 3 groups; the reference gene for humans was identified in mice models; no evaluation of the target genes
(Jimenez-Ortega et al., 2017)	PM Mexican-Mestizo women: 6 with normal (control group) and 6 with OP hip BMD (age 63-85 years)	PBMCs	Histopaque-1077 kit used for obtaining PCMCs by density gradients. CD14+ obtained by density gradient centrifugation for 30 min at 400g and RT and magnetic bead isolation. negative isolation kit EasySep Human Monocyte Enrichment used for naive monocyte isolation	Screening: Affymetrix GeneChip Human U133 Plus 2.0 Array Validation: TaqMan RT-qPCR	Screening: 2.578 miRNAs tested Validation: miR-1270, miR-548x-3p, and miR-8084	Screening: quantile normalization Validation: RNU44	↑miR-1270 in OP group vs. control group	IRF8 (identified for miR-1270 using PITA v5.0, microRNA.org, miRWalk v2.0, miRDB, and TargetScan Human v7.0 database and validated in study)	/	Small sample size; no ROC analysis
(De-La-Cruz-Montoya et al., 2018)	PM Mexican-Mestizo women: 7 with normal (control group) and 7 with OP hip BMD (age 63-85 years)	Human PBMCs	Blood collected in CPT tubes and PBMCs obtained. CD14+ cells enriched by negative selection (EasySep kit)	Screening: Illumia NextSeq 500 Validation: TaqMan RT-qPCR	Validation: miR-708-5p, miR-3161, miR-939-3p, and miR-4422	Validation: RNU44 and RNU48	↑miR-708-5p in osteoporosis group vs. control group	AKT1, AKT2, FKBP5, PARP1, and MP2K3 (identified for miR-708-5p using miRTarBase and MiRNet and validated in study)	/	Small sample size; no ROC analysis
(Ramirez-Salazar et al., 2018)	Discovery cohort: 40 PM Mexican-Mestizo women: 20 with normal (controls) and 20 with OP hip BMD (age 63-85 years)Validation cohort: 22 normal, 26 OP, 28 osteopenia, 21 with hip fracture BMD	Cell-free serum	Serum obtained within 1h of collection and stored at -80°C	Discovery stage: TaqMan Array Human MicroRNA A+B Cards Set v3.0 Validation stage: TaqMan RT-qPCR	Screening: 754 miRNAs tested Validation: miR-23b-3p miR-140-3p, and miR-885-5p	snRNU6	↑ miR-23b-3p and miR-140-3p in OP, osteopenia and bone fracture group vs. control↑ miR-885-5p in osteopenia vs. control	AKT1, AKT2, AKT3, IL6R, BMP2, GSK3B, FOXO3, PRKACB, WNT5B, and RUNX2 (identified for miR-23b-3p and miR-140-3p using miRWalk v3 database)	0.84 (miR-140-3p) for osteopenia, 0.96 (miR-140-3p) for OP, and 0.92 (miR-140-3p) for fracture vs. HC0.73 (miR-23b-3p) for osteopenia, 0.69 (miR-23b-3p) for OP, and 0.88 (miR-23b-3p) for fracture vs. HC0.69 (miR-885-5p) for osteopenia vs. HC	No validation of the identified target genes

AKT1, AKT serine/threonine kinase 1; AKT2, AKT serine/threonine kinase 2; AKT3, AKT serine/threonine kinase 3; BMD, bone mineral density; BM-MCSs, bone marrow mesenchymal stem cells; BMP2, bone morphogenic protein 2; CBL, casitas B-lineage lymphoma proto oncogene; CD226, cluster of differentiation 226; CXCL11, chemokine (C-X-C motif) ligand 11; CXCR3, chemokine (C-X-C motif) receptor 3; FKBP5, FK506 binding protein 5; FOXO3, forkhead box O3; FZD3, frizzled-3; GSK3B, glycogen synthase kinase 3 beta; HC, healthy controls; IGF1, insulin-like growth factor 1; IL6R, interleukin 6 receptor; IRF8, interferon regulatory factor-8; Mef2c, myocyte enhancer factor 2 c; MP2K3, mitogen-activated protein kinase kinase 3; OP, osteoporosis; OSX, osterix; PAG1, phosphoprotein associated with glycosphingolipid microdomains 1; PARP1, poly(ADP-ribose) polymerase 1; PBMCs, peripheral blood mononuclear cells; PM, postmenopausal; PRKACB, protein kinase cAMP-activated catalytic subunit beta; RANK, receptor activator of nuclear factor κ B; RANKL, receptor activator of nuclear factor k B ligand; RT, room temperature; RT-qPCR, real-time quantitative polymerase chain reaction; RUNX2, runt-related transcription factor 2; SLC39A1, solute carrier family (zinc transporter), member 1; SPRY1, protein sprouty homolog 1; TOB2, transducer of ERBB2, 2; WNT5B, Wnt family member 5B.

### miRNAs, Bone Fragility, and Bone Fracture Risk in Postmenopausal Women

Bone fragility and fractures are the clinically relevant consequences of OP and have a negative impact on quality of life. Considering the objective limit of bone biopsy in healthy individuals, studies have compared the miRNA expression profile of OP bone with osteoarthritis (OA) samples as control. Thirteen of 760 miRNAs assayed by microarray cards were found differentially expressed in bone specimens from the femur heads of eight women with OP hip fracture compared to the femur heads from eight women with severe hip OA but without OP hip fracture, in seven of which the miRNAs were overexpressed in OP bones. In the following replication stage, the results showed that miR-518f was overexpressed and miR-187 downregulated in OP compared with OA bone (Garmilla-Ezquerra et al., 2015). Finally, the expression profile of 1,932 miRNAs was compared between fresh femoral neck trabecular bone from postmenopausal women with OP hip fracture and from postmenopausal women with OA non-OP hip fracture (control group). Following validation, only two (miR-320a and miR-483-5p) of the 82 miRNAs differently expressed between the two groups were significantly overexpressed in the OP vs. the OA samples (De-Ugarte et al., 2015). miRNA-320a targets RUNX2 and β-catenin (Yu et al., 2011; Sun et al., 2012), while miRNA-483-5p downregulates IGF2 expression in OP-derived human osteoblast cultures (De-Ugarte et al., 2015).

To identify circulating miRNAs as biomarkers for OP fracture, Seeliger et al. (2014) assayed a panel of 83 serum miRNAs in OP and non-OP patients with either femoral neck or pertrochanteric fracture. Eleven miRNAs (miR-100-5p, miR-122a-5p, miR-124-3p, miR-125b-5p, miR-148a-3p, miR-21-5p, miR-223-3p, miR-23-3p, miR-24-3p, miR-25-3p, and miR-27a-3p) were found at significantly higher levels in the OP sera. Together with miR-93 and miR-637, these miRNAs were subsequently validated in another set of serum samples: nine miRNAs (miR-100, miR-122a, miR-124a, miR-125b, miR-148a, miR-21, miR-23a, miR-24, and miR-93) were significantly higher in the OP sera than in the controls and they were proposed as markers to differentiate OP from non-OP bone fracture. Interestingly, miR-21 was previously found downregulated in both the BM-MCSs and the plasma of OP patients (Yang et al., 2013; Li et al., 2014); these opposite results could be ascribed to the different experimental protocols used, which identified miRNAs that regulate osteoclast/osteoblast differentiation and activity, as previously demonstrated. miR-21 is highly expressed in osteoclast precursors and it is upregulated in the course of TNF-α/RANKL-induced osteoclastogenesis (Fujita et al., 2008; Kagiya and Nakamura, 2013); it stimulates osteoclastogenesis by overcoming PDCD4-mediated c-Fos inhibition (Fujita et al., 2008; Sugatani et al., 2011), while its expression is inhibited by estrogens (Garcia Palacios et al., 2005; Sugatani and Hruska, 2013). miR-23 and miR-24 belong to the miR-23a∼27a∼24-2 cluster and act as negative regulators of osteoblast differentiation by targeting SATB2 that cooperates with RUNX2 to induce osteogenesis, while miR-23a also inhibits RUNX2 (Hassan et al., 2010). miR-93 inhibits osteoblast mineralization by targeting OSX (Yang et al., 2012). miR-100 negatively regulates BMPR2, a key osteogenic factor for MSCs (Zeng et al., 2012). The overexpression of miR-125b is associated with impaired osteoblast differentiation and proliferation through the modulation of OSX expression (Mizuno et al., 2008; Chen et al., 2014b). miR-124 is progressively downregulated during RANKL-induced osteoclastogenesis and its overexpression affects the maturation of osteoclast precursors *via* suppression of the key osteoclastogenic factor NFATc1, and their migration *via* inhibition of RhoA/Rac1 (Lee et al., 2013).

Following the identification of nine miRNAs whose circulating levels were higher in OP patients than in controls, Seelinger et al. evaluated their expression in the bone tissues: miR-100, miR-125b, miR-21, miR-23a, miR-24, and miR-25 were upregulated also in the OP bone samples. They defined the potential diagnostic value of these miRNAs by means of ROC curve analysis. All the identified serum miRNAs showed significant AUC, sensitivity and specificity in discriminating OP from non-OP subjects: 0.69–62.9%–61.7% (miR‐100), 0.77–74.1%–72.1% (miR‐122a), 0.69–61.4%–61.0% (miR‐124a), 0.76–76.4%–75.0% (miR‐125b), 0.61–62.5%–62.3% (miR‐148a), 0.63–61.3%–61.7% (miR‐21), 0.63–57.4%–56.7% (miR‐23a), 0.63–60.3%–60.4% (miR‐24), and 0.68–69.0%–68.3% (miR‐93). Consequently, the five miRNAs identified in both tissue and serum samples can be used as biomarkers for OP and related hip fractures (Seeliger et al., 2014).

Another study attempted to search for potential miRNAs marking for OP bone fractures. In the discovery stage, Caucasian women with either OP sub-capital hip fracture (n = 8) or severe hip OA (control group, n = 5), which required arthroplasty, were recruited (Panach et al., 2015). The serum levels of 179 miRNAs were analyzed by RT-qPCR. Among the 42 differently regulated miRNAs, six (miR-122-5p, miR-125b-5p, miR-143-3p, miR-21-5p, miR-210, and miR-34a-5p) were selected for the replication stage. miR-122-5p, miR-125b-5p, and miR-21-5p were significantly higher in the OP bone fracture group than the controls. miR-125b-5p and miR-21-5p have been correlated with bone metabolic indexes (Fujita et al., 2008; Mizuno et al., 2008; Sugatani and Hruska, 2013), and the upregulation of miR-21 was consistent with previous observations (Seeliger et al., 2014). ROC analysis of the diagnostic value of the serum miRNAs revealed that miR-122-5p, miR-125b-5p, and miR-21-5p consistently discriminated between the OP patients with fractures (n = 15) and the controls (n = 12) (AUC 0.87 for miR-122-5p, 0.76 for miR-125-5p, and 0.87 for miR-21-5p) (Panach et al., 2015). Using a similar protocol, Weilner et al. (2015) found three other miRNAs potentially correlated with OP fractures in postmenopausal women (n = 7 in the discovery stage, n = 12 in the validation stage) (miR-22-3p, miR-328-3p, and let-7g-5p) and that the levels were significantly lower in the serum of the cases (n = 7 in the discovery stage, n = 11 in the validation stage). Previous *in vitro* experiments demonstrated that let-7 promotes osteoblastogenesis in MSCs *in vitro*, while it induces bone formation *in vivo*. These effects are mediated by the repression of high-mobility group AT-hook 2 (HMGA2) (Wei et al., 2014). *In vitro* experiments on human unrestricted somatic stem cells (USSC) showed that miR-22-3p is upregulated during osteogenic differentiation and that its potential target is CDK6 (Trompeter et al., 2013). Finally, CD44 is a potential target of miRNA-328-3p in macrophages and it is also expressed in osteocytes (Ishimoto et al., 2014). *In vitro* experiments on MSCs collected from two OP patients with bone fracture confirmed the let-7g-5p-mediated effect and miR-22-3p downregulation, and correlated miR-328-3p repression with reduced ALP activity during osteogenic formation (Weilner et al., 2015).

Recent studies have investigated whether single or combined miRNAs discriminate bone fractures in conditions associated with bone fragility. Kocijan et al. (2016) performed a case-control study to identify serum miRNAs correlated with trauma fractures in postmenopausal OP. Three (miR-152-3p, miR-320a, and miR-335-5p) of the 187 tested miRNAs selected based on previously published studies were significantly higher, whereas sixteen (let-7b-5p, miR-140-5p, miR-16-5p, miR-186-5p, miR-19a-3p, miR-19b-3p, miR-215-5p, miR-29b-3p, miR-30e-5p, miR-324-3p, miR-365a-3p, miR-378a-5p, miR-532-5p, miR-550a-3p, miR-7-5p, and miR-93-5p) were significantly lower in postmenopausal women with bone fracture (n = 10) than in the controls without bone fracture (n = 11). ROC analysis showed that miR-140-5p, miR-152-3p, miR-19a-3p, miR-19b-3p, miR-30e-5p, miR-324-3p, miR-335-5p, and miR-550a-3p had a higher discriminating power between individuals with bone fracture and healthy individuals (AUC> 0.9) than BMD or bone turnover markers. miR-335-3p has been reported to promote osteogenic differentiation by binding and downregulating dickkopf-related protein 1 (DKK1), a soluble antagonist of the Wnt signaling pathway (Zhang et al., 2011a). miR-30e has been reported to be downregulated during osteoblastic differentiation of MSC, and its target has been identified in low-density lipoprotein receptor-related protein 6 (LRP6), a known critical factor in Wnt signaling (Wang et al., 2013b). miR-140-5p inhibits osteoblastic differentiation of hMSCs by repressing bone morphogenic protein 2 (BMP2) (Hwang et al., 2014). miR-29 family members (miR-29a-3p, miR-29b-3p, and miR-29c-3p) are upregulated during osteoclastogenesis, while their KO results in altered recruitment and migration of osteoclast precursors without any effect on osteoclast activity (Franceschetti et al., 2013). In addition, six targets (Cdc42, srGAP2, GPR85, NFIA, CD93, and CTR) of the miR-29 family are involved in cytoskeletal organization, recruitment of osteoclast precursors, and osteoclast function (Franceschetti et al., 2013). However, results for miR-29 family roles are conflicting. The administration of pre-miR-29a in rats limited the bone loss induced by glucocorticoids, while miR-29b expression was downregulated during the differentiation of CD14+ PBMCs into osteoclasts (Rossi et al., 2013; Wang et al., 2013a). These effects are probably associated with the miR-29 family action on Wnt signaling and on osteoblast activity promotion (Wang et al., 2013a). In another study, miR-29b resulted upregulated in RAW264.7 cells treated with TNF-α and RANKL to induce osteoclastogenesis (Kagiya and Nakamura, 2013). Furthermore, miR-29b has been found to promote osteogenesis and to regulate extracellular matrix proteins expression by targeting the expression of HDAC4, TGF3, ACVR2A, CTNNBIP1, DUSP2 and COL1A1, COL5A3, COL4A2, respectively (Li et al., 2009).

Recent studies have discovered other circulating miRNAs associated with OP and OP bone fracture. Chen et al. (2017) tried to find other potential serum and tissue miRNAs in Chinese OP women with hip fractures. Five of the 95 detected miRNAs were significantly upregulated in the OP patients (n = 30) compared with the healthy non-OP controls (n = 30): miR-125b, miR-30, miR-4665-3p, miR-5914, and miR-96. Only miR-125b, miR-30, and miR-5914 were subsequently validated by RT-qPCR. These three miRNAs were also found upregulated in OP bone samples compared with controls. In both cases, miR-125b was the most upregulated, and ROC analysis confirmed its diagnostic potential in postmenopausal OP (AUC 0.898) in accordance with three previous studies (Seeliger et al., 2014; Panach et al., 2015; Kelch et al., 2017).

Yavropoulou et al. (2017) investigated the expression level of fourteen serum miRNAs, previously associated with OP and OP bone fractures in the sera from postmenopausal women with low bone mass and either with (n = 35) or without (n = 35) vertebral fractures. Compared with the controls, miR-124-3p and miR-2861 were higher, whereas miR-21-5p, miR-23a-3p, and miR-29a-3p were lower in the two OP groups compared with the non-OP controls. Furthermore, in the patients with low bone mass, the levels of miR-21-5p were lowest in the patients with vertebral fractures. Together with their above- described role, miR-124-3p, miR-21-5p, miR-23a-3p, miR-2861, and miR-29a-3p are known to positively regulate osteoblast differentiation by targeting HDAC5, a transcriptional factor that affects bone formation mediated by Runx2 (Hu et al., 2011). ROC analysis showed that the associated AUC of miR-21-5p was 0.66, with 66% sensitivity and 71% specificity (Yavropoulou et al., 2017). These results contrasted with those from previous studies that found an association between miR-21-5p and miR-23-3p upregulation with bone fractures in OP (Seeliger et al., 2014; Panach et al., 2015; Kelch et al., 2017). Wang et al. (2018) identified eight out of ten miRNAs in sera and bone tissue samples from OP patients with bone fracture. miR-100, miR-122a, miR-125b, miR-24-3p, and miR-27a-3p levels were higher in serum and upregulated in the bone samples of OP patients (n = 45) than in the non-OP subjects (n = 15), while miR-128 was upregulated only in the OP bone samples. Conversely, miR-145 expression was increased only in the OP serum compared with non-OP, while miR-144-3p was downregulated in the OP serum and the bone samples. Since miR-144-3p has not been associated with OP, the authors further investigated its role in osteoclastogenesis. miR-144 was found to affect osteoclast differentiation by targeting RANK, as well as proliferation and apoptosis.

Recently, Li et al. (2018) conducted a study to validate serum miR-133a as a biomarker for postmenopausal OP with bone fracture. miR-133a upregulation in circulating monocytes and in serum has been associated with postmenopausal OP (Wang et al., 2012; Li et al., 2014). The study reported that serum miR-133a was significantly higher in the postmenopausal OP women with hip fracture than in the healthy controls, and that it negatively correlated with BMD at the lumbar spine. *In vitro*, miR-133a expression was significantly upregulated during RANKL/M-CSF-induced osteoclastogenesis in RAW264.7 and THP-1 cells and its overexpression upregulated NFATc1, c-Fos, and TRAP protein expression (Li et al., 2018). Previous studies have also demonstrated that miR-133a overexpression in the osteoblast cell line MC3T3 suppressed osteoclastogenesis by directly targeting RUNX2 (Zhang et al., 2011b). *In vivo*, miR-133a KO in OVX rats altered the circulating levels of osteoclastogenesis-related factors and prevented bone loss (Li et al., 2018). Taken together, these findings support the diagnostic potential for miR-133a in postmenopausal OP and related bone fracture and highlight the potential of miR-133a as a clinical therapeutic target for postmenopausal OP.

**Table 2** summarizes information about circulating miRNAs associated with bone fracture risk in OP.

**Table 2:** miRNAs related to bone fracture risk in postmenopausal OP.

Study	Study design	Biomarker source	Sample handling	Quantification platform	Evaluated miRNA	Normalization strategy	Reported miRNA biomarker	Potential target gene	AUC-Sensitivity (%)-Specificity (%)	Limits
(Garmilla-Ezquerra et al., 2015)	Discovery cohort: 8 women with OP hip fracture, 8 women with severe hip OA without OP fractures (control group) Replication cohort: 19 women with OP hip fracture, 19 women with severe hip OA without OP fractures (control group)	Bone specimens	Trabecular bone cylinders obtained from central part of femoral head using a trephine. Fragments cut into small pieces, washed with PBS, snap-frozen in liquid nitrogen, and stored at -70°C	Discovery stage: TaqMan array human miRNA A + B cards v3 Replication stage: TaqMan RT-qPCR	Discovery stage: 760 miRNAs tested Replication stage: miR-187, miR-193a-3p, miR-214, miR-518f, miR-636, and miR-210	NormFinder and GeNorm programs identified miR-222 and let-7b as most stable normalizators.	↑ miR-518f in OP fractures group vs. control group↑ miR-187 in control group vs. OP fractures group	IGFBP1, DKK1, WISP1, CTNNBIP1 (identified for miR-518f using microRNA.org, mirbase.org, and targetscan.org prediction algorithms but not validated by *in vitro* experiments)	/	Small sample size; OA patients as control group; no validation of the identified target genes; no information about the stem-loop arm of miRNA origin; no ROC analysis.
(De-Ugarte et al., 2015)	Discovery cohort: 6 PM OP women and 6 PM OA women (control group) both with femoral neck fracture Replication cohort: 7 PM OP women and 6 PM OA women (control group) both with femoral neck fracture	Fresh bone specimens	Bone fragments from femoral neck transcervical region reduced to small pieces, washed three times with PBS, and stored at -80°C	Discovery stage: miRCURY LNA™ microRNA Array performed by Exiqon Services Replication stage: RT-qPCR performed by Exiqon Services	Discovery stage: 1932 miRNAs tested Replication stage: miR-675-5p, miR-30c-1-3p, miR-483-5p, miR-542-5p, miR-142-3p, miR-223-3p, miR-32-3p, and miR-320a	Discovery stage: Lowess (Locally Weighted Scatterplot Smoothing) global regression algorithm. Replication stage: average of miR-let-7e-5p expression in each sample	↑ miR-320a and miR-483-5p in OP fractures vs. control group	ARPP-19, BMP3 and 6, BMPR1A, CAMTA1, DNER, ESRRG, IGF1, IGF1R, IL6R, JAK2, PPARGC1A, LEPR, MAPK1, MCL, NR3C1, PDGFD, PTGER3, RARG, RXRA, SGK, SP1, SRF, TFR1 (identified for miR-320a using PicTar, TargetScan Human, miRDB, MiRanda, DIANA-TarBase, and miRTarBase database)SRF and MAPK3 (identified for miR-483-5p using mirTArBase)	/	Small sample size; OA patients as control group; no validation of the identified target genes; no ROC analysis
(Seeliger et al., 2014)	Discovery cohort: 10 OP (7 women and 3 men) and 10 non-OP (10 women) as control group, both with femoral neck or pertrochanteric fracture Replication cohort: 30 OP women and 30 non-OP women (control group), both with femoral neck or pertrochanteric fracture	Discovery stage: cell-free serum Replication stage: cell-free serum and bone tissue	/	Screening: human Serum & Plasma miRNA PCR Array MIHS-106Z Validation: SYBR RT-qPCR	Screening: 83 miRNAs tested Validation: miR-21-5p, miR-23-3p, miR-24-3p, miR-25-3p, miR-27a-3p, miR-93, miR-100-5p, miR-122a-5p, miR-124-3p, miR-125b-5p, miR-148a-3p, miR-223-3p, and miR-637	Average of SNORD96a and snRNU6	↑ miR-21, miR-23a, miR-24, miR-93, miR-100, miR-122a, miR-124a, miR-125b, and miR-148a in OP fracture serum vs. controls↑ miR-21, miR-23a, miR-24, miR-25, miR-100, and miR-125b in bone tissue from OP fracture patients vs. control	PDCD4, cFos (miR-21); RUNX2 (miR-23a/miR-24-2/miR-27a complex); OSX (miR-93); BMPR2 (miR-100); VCAN (miR-124a); RANKL (miR-148a)(identified from previous papers but not validated in this paper)	0.63-61.3-61.7 (miR-21), 0.63-57.4-56.7 (miR-23a), 0.63-60.3-60.4 (miR-24), 0.68-69.0-68.3 (miR-93), 0.69-62.9-61.7 (miR-100), 0.77-74.1-72.1 (miR-122a), 0.69-61.4-61.0 (miR-124a), 0.76-76.4-75.0 (miR-125b), 0.61-62.5-62.3 (miR-148a) for OP fracture vs. non-OP	Small sample size; no validation of the target genes.
(Panach et al., 2015)	Discovery stage: 8 Caucasian women with OP subcapital hip fracture and 5 with severe OA of hip requiring surgery (control group)Replication stage: 15 Caucasian women with OP subcapital hip fracture and 12 with severe OA of hip requiring surgery (control group)	Cell-free serum	Serum samples obtained from fasting blood stored at -80°C	Discovery stage: miRCURY LNA Universal RT microRNA PCR, Serum/Plasma Focus microRNA PCR Panel Replication stage: Exiqon LNA RT-qPCR	Screening: 179 miRNAs tested Validation: miR-143-3p, miR-122-5p, miR-125b-5p, miR-210, miR-21-5p, and miR-34a-5p	GeNorm identified miR-93-5p	↑ miR-122-5p, miR-125b-5p, and miR-21-5p in OP fracture vs. control group	/	0.87 (miR-122-5p), 0.76 (miR-125-5p), and 0.87 (miR-21-5p) for OP fracture vs. control group	Small sample size, OA patients as control group; no evaluation of the target genes
(Weilner et al., 2015)	Discovery stage: 7 PM Caucasian women with femoral neck OP fracture and 7 PM women without femoral fracture (control group)Replication stage: 12 PM Caucasian women with femoral neck OP fracture and 11 PM women without femoral fracture (control group)	Cell-free serum	Serum obtained from blood samples centrifugied at RT and 2000g for 15 min, after incubation at RT for 30 min, and stored at -80°C	Screening: Exiqon serum/plasma focus panels Validation: RT-qPCR	Screening: 175 miRNAs tested Validation: miR-10a-5p, miR-10b-5p, miR-22-3p, miR 133b, miR-328-3p, and let-7g-5p	Normalization of Cp-values based on average Cp of the detected miRNAs	↓ miR-22-3p, miR-328-3p, and let-7g-5p in OP fracture serum vs. control group	/	/	Small sample size; no evaluation of the target genes; the mean age of patients recruited for the discovery and validation study was significantly different (71 years and 80 years, respectively); no ROC analysis
(Kocijan et al., 2016)	10 women with PM OP low trauma fracture and 11 healthy PM women without low-trauma fracture	Cell-free serum	Fasting blood samples immediately centrifuged and serum stored a -80°C	SYBR Green RT-qPCR	187 miRNAs tested	Global mean	↑ miR-152-3p, miR-335-5p, miR-320a and↓ let-7b-5p, miR-7-5p, miR-16-5p, miR-19a-3p, miR-19b-3p, miR-29b-3p, miR-30e-5p, miR-93-5p, miR-140-5p, miR-215-5p, miR-186-5p, miR-324-3p, miR-365a-3p, miR-378a-5p, miR-532-5p, and miR-550a-3p in fractured group vs. control group	/	0.962 (miR-152-3p), 0.959 (miR-30e-5p), 0.950 (miR-324-3p), 0.947(miR-140-5p), 0.944 (miR-19b-3p), 0.939 (miR-335-5p), 0.929 (miR-19a-3p), 0.909 (miR-550a-3p), 0.898 (miR-186-5p), 0.898 (miR-532-5p), 0.872 (miR-378a-5p), 0.870 (miR-320a), 0.879 (miR-93-5p), 0.857 (miR-16-5p), 0.853 (miR-215-5p), 0.852 (let-7b-5p), 0.824 (miR-7-5p), 0.838 (miR-29b-3p), and 0.809 (miR-365a-3p) for fracture group vs. control group	Small sample size; no evaluation of the target genes; arbitrary choice of the screened miRNAs
(Chen et al., 2017)	30 PM Chinese women with OP and 30 PM Chinese women without OP (control group) both with hip fracture	Cell-free serum and bone tissues	Blood samples allowed to clot, centrifuged at 1500g, then serum isolated and stored.	Screening: Microarray Validation: TaqMan RT-qPCR	Validation: miR-30, miR- 96, miR-125b, miR-4665-3p, and miR-5914	snRNU6	↑ miR-125b, miR-30 and miR-5914 in serum and bone tissues from OP fracture vs. control group	/	0.699 (miR-5914), 0.757 (miR-30), and 0.898 (miR-125b) for OP fracture vs. controls	Small sample size; no target genes evaluation; no information about the stem-loop arm of miRNA origin
(Yavropoulou et al., 2017)	35 PM women with low bone mass without vertebral fractures, 35 with low bone mass and vertebral fractures, 30 HC	Cell-free serum	Blood samples collected in clot activator tubes, placed at RT for 10-60 min, centrifuged for 10 min at 1900g and 4°C. Serum samples centrifuged again for 10 min at 16000g and 4°C and frozen at -80°C	SYBR Green RT-qPCR	14 miRNAs selected based on the existing literature: miR-21-5p, miR-23a-3p, miR-24-2-5p, miR-26a-5p, miR-29a, miR-33a-5p, miR-124-3p, miR-133a, miR-135b-5p, miR-214-3p, miR-218-5p, miR-335-3p, miR-422, and miR-2861	Panel of SNORD95, SNORD96A, and snRNU6-2	↑ miR-124-3p, miR-2861, and ↓ miR-21-5p, miR-23a-3p, miR-29a-3p in OP vs. controls↓miR-21-5p in OP with vertebral fracture vs. OP without vertebral fracture	SPRY1, BMP3, DKK2, and SMAD7 (miR-21-5p); SATB2 and RUNX2 (miR-23a-3p); SATB2 and CALB1 (miR-24-2-5p); EPHA5, COL10A1, and COL19A1 (miR-26a-5p); DUSP2, COL3A1, COL5A3, and PTHLH (miR-29a); DKK2, WIF1, and OSTF1 (miR-33a-5p); HDAC5, NFATC1, and NFATC2, (miR-124-3p); ACVR1B, FOXO1, SIRT1, and SMAD5 (miR-135b-5p); ATP2A3, CTNNB1, and VDR (miR-214-3p); COL1A1, SFRP2, SOST, and EPHA5 (miR-218-5p); DKK1 and SPARC (miR-335-3p); HDAC5 (miR-2861)(Identified using miRBase, DIANA TOOLS, PicTar, miRDB, TargetScanHuman, miRGator, and microRNA database)	0.66-66-71 (miR-21-5p) for OP with vertebral fracture vs. OP without vertebral fracture	Small sample size; no validation of the identified target genes
(Wang et al., 2018)	45 OP patients, 15 non-OP (control group) both with femoral fracture	Cell-free serum and bone tissues	/	RT-qPCR	miR-7-5p, miR-24-3p, miR-27a-3p, miR-100, miR-125b, miR-128, miR-145-5p, miR-211-5p, miR-144-3p, and miR-122a	snRNU6	↑ miR-24-3p, 27a-3p, miR-100, miR-125b, miR-122a, miR-145, and ↓ miR-144-3p in serum from OP fracture vs. non-OP fracture↑ miR-24-3p, 27a-3p, miR-100, miR-125b, miR-128, miR-122a, and ↓ miR-144-3p in bone tissues form OP fracture vs. non-OP fracture	RANK (identified for miR-144-3p using TargetScan online software and validated by *in vitro* study)	/	Small sample size of the non-OP group; no ROC analysis
(Li et al., 2018)	10 PM Chinese OP women with hip fracture and 10 HC	Cell-free serum	Blood samples allowed to clot then centrifuged at 1500g to obtain serum	TaqMan RT-qPCR	miR-133a	snRNU6	↑miR-133a in OP with fractures vs. HC	c-Fos, NFATc1, and TRAP for miR-133a identified by *in vitro* experiments	/	Small sample size; no ROC analysis

ACVR1B, activin A receptor type 1B; ALPL, alkaline phosphatase; ANKH, ANKH inorganic pyrophosphate transport regulator; AR, androgen receptor; ARPP-19, cAMP-regulated phosphoprotein 19; ATF4, activating transcription factor 4; ATP2A3, sarcoplasmic/endoplasmic reticulum calcium ATPase 3; BMP2K, BMP2 inducible kinase; BMP3, bone morphogenetic protein 3; BMP6, bone morphogenetic protein 6; BMPR1A, bone morphogenetic protein receptor 1A; BMPR2, bone morphogenetic protein receptor type 2; CALB1, calbindin 1; CAMTA1, calmodulin binding transcription activator 1; CNR1, cannabinoid receptor 1; CNR2, cannabinoid receptor 2; COL10A1, collagen type X alpha 1 chain; COL19A1, collagen type XIX alpha 1 chain; COL1A1, collagen type I alpha 1 chain; COL3A1, collagen type III alpha 1 chain; COL5A3, collagen type V alpha 3 chain; CTNNB1, catenin beta 1, CTNNBIP1, catenin-interacting protein 1; DKK1, Dickkopf WNT signaling pathway inhibitor 1; DKK2, Dickkopf WNT signaling pathway inhibitor 2; DNER, delta and notch-like epidermal growth factor-related receptor; DUSP2, dual specificity phosphatase 2; EPHA5, EPH receptor A5; ESR1, estrogen receptor 1; ESRRG, estrogen related receptor gamma; FOXO1, Forkhead box O1, FSHB, follicle stimulating hormone subunit beta; HC, healthy controls; HDAC5, histone deacetylase 5; IGF1, insulin-like growth factor; IGF1R, insulin-like growth factor 1 receptor; IGFBP1, insulin-like growth factor binding protein 1; IL6R, interleukin 6 receptor, JAK2,Janus kinase 2; LEPR, leptin receptor; LRP6, LDL receptor related protein 6; MAPK1, mitogen-activated protein kinase 1; MAPK3, mitogen-activated protein kinase 3; MCL, myeloid cell leukemia; MSCs, mesenchymal stem cells; NFATC1, nuclear factor of activated T cells 1; NFATC2, nuclear factor of activated T cells 2; NR3C1, nuclear receptor subfamily 3 group C member 1; OA, osteoarthritis; OP, osteoporosis; OSTF1, osteoclast stimulating factor 1; OSX, osterix; PDCD4, programmed cell death 4; PDGFD, platelet-derived growth factor D; PM, postmenopausal; PPARGC1A, peroxisome proliferator-activated receptor gamma coactivator 1-alpha; PTGER3, prostaglandin E receptor 3; PTHLH, parathyroid hormone like hormone; RANKL, receptor activator of nuclear factor k B ligand; RARG, retinoic acid receptor gamma; RT, room temperature; RT-qPCR, real-time quantitative polymerase chain reaction; RUNX2, runt-related transcription factor 2; RXRA, retinoid X receptor alpha; SATB2, SATB homeobox 2; SFRP2, secreted frizzled related protein 2; SGK, serine/threonine protein-kinase; SIRT1, sirtuin 1; SMAD5, SMAD family member 5; SMAD7, SMAD family member 7; SOST, sclerostin; SPARC, secreted protein acidic and cysteine rich; SPRY1, protein sprouty homolog 1; SRF, serum response factor; T2DM, Type 2 diabetes mellitus; TFR1, transferrin receptor protein 1; TRAP, triiodothyronine receptor auxiliary protein; TSC22D3, TSC22 domain family member 3; VCAN, versican; VDR, vitamin D receptor; WIF1, WNT inhibitory factor 1;WISP1, WNT1-inducible-signaling pathway protein 1.

### miRNAs, Fracture Risk, and Physical Activity

Physical activity (PA) is a therapeutic strategy to reduce bone fracture risk, improve bone metabolic status and, eventually, to increase bone mass during childhood, adolescence, and early adulthood or to limit the age-associated decrease in peak bone mass in older age (Xu et al., 2016). PA affects miRNAs expression in tissues and organs, the circulating miRNAs profile reflects this situation as a consequence (Lombardi et al., 2016a). The literature on PA-dependent modifications of osteoporosis- or fracture risk-associated miRNAs is scarce (Lombardi et al., 2016a). The suboptimal understanding of these mechanisms stems from failure to appreciate the complex network of interactions accompanying the metabolic response of bone to PA. This multilevel relationship contemplates: direct effects of PA on bone; whole-body metabolic effects of PA on bone; specific effects of PA on tissues (e.g., skeletal muscle, adipose tissue, immune system, nervous system) besides the release of mediators from bone (e.g., myokines, adipokines, cytokines, and neurotransmitters) that affect bone both directly and indirectly; and PA-dependent release of mediators by bone (osteokines) that affect the expression of bone-acting mediators released by other tissues (Lombardi et al., 2016b; Lombardi, 2019). Recently, we demonstrated that seven from a panel of ten fracture risk-associated miRNAs (miR-100, miR-122-5p, miR-125-5p, miR148a-3p, miR-23a-3p, miR-24-3p, and miR-93-5p) responded to a protocol of PA (8-week repeated sprint training in young healthy males) in a more sensitive way than standard bone metabolism markers, metabolic hormones, and cytokines (Sansoni et al., 2018).

### miRNAs in Other Types of OP and Related Fracture Risk

Considering senile OP, a study investigated the role of a specific miRNA (miR-125b) in osteoblast differentiation (Chen et al., 2014b). miR-125b was selected due to its crucial involvement in the epigenetic regulation of proliferation/differentiation of cell lineages (Liu et al., 2011). miR-125b expression levels in BM-MSCs was found upregulated in small mixed gender populations of senile Chinese OP patients (n = 4, 3 women and 1 man) compared with subjects with normal BMD (control group, n = 5, 2 women and 3 men). miR-125b upregulation was associated with impaired BM-MSCs proliferation and osteogenic differentiation and, consistent with these observations, the antagonism of miR-125b in non-OP BM-MSCs promoted proliferation, osteoblast differentiation, and mineralization. In these cells, miR-125b also targeted Osterix (OSX), a key transcription factor for osteogenic differentiation (Chen et al., 2014b). Weilner et al. (2016) found that the presence of miR-31 in circulating microvesicles derived from senescent endothelial cells negatively impacted on the osteogenic differentiation capacity of adipose tissue-derived MSCs. Circulating miR-31 levels were higher in the plasma samples from elderly healthy donors than in young healthy controls, as well as in the plasma from OP patients compared with healthy age-matched controls. miR-31 directly inhibits osteoblast formation by targeting Frizzled-3 (FZD3). Also SATB2, Osx, and RUNX2 have been validated as targets of miR-31 (Baglio et al., 2013; Deng et al., 2014; Xie et al., 2014). This miRNA is involved in osteoclastogenesis: its expression has been found strongly upregulated during RANKL-induced osteoclast differentiation and its inhibition by specific antagomirs results in impaired osteoclast differentiation, actin ring formation, and bone resorption (Mizoguchi et al., 2013). These alterations depend upon the overexpression of the miR-31 target gene RhoA, a GTPase involved in the transduction of extracellular signals to the cytoskeleton (Mizoguchi et al., 2013). This study showed, for the first time, that the miRNA content from senescent cells-derived microvesicles might correlate with the impairment of bone formation and that miR-31 can be used as a biomarker for age-associated diseases such as OP (Weilner et al., 2016). Nonetheless, a larger cohort is needed to confirm these data.

Studies have attempted to correlate circulating and tissue-altered miRNAs expression with the risk of bone fracture in senile OP patients. In bone tissue samples from elderly Chinese patients with bone fracture, miRNA quantification by RT-PCR revealed that miR-214 expression correlated positively with age and negatively with bone formation marker levels (osteocalcin and alkaline phosphatases) (Wang et al., 2013c). The major limitations of the study were: small sample size, unclear comparison between aged and control groups, and missing information about the screened miRNAs and data normalization. In murine pre-osteoblast MC3T3-E1 cells, miR-214 negatively affected osteoblast activity and matrix mineralization by targeting activating transcription factor 4 (ATF4); these features were restored by antagomiR-214 and further accentuated by agomiR-214. Furthermore, miR-214 inhibition improved the bone phenotype in OVX and hind limb-unloaded mice, whereas osteoblast activity was limited and bone mass reduced in miR-214 transgenic mice (Wang et al., 2013c). In 2017, the nine serum miRNAs associated with OP found by Seeliger et al. (2014) were validated also in serum, bone specimens, and cultured osteoblasts and osteoclasts from another cohort of OP (n = 14, 7 women and 7 men) and OA patients (n = 14, 7 women and 7 men) with hip fractures (Kelch et al., 2017). The expression levels of miR-100-5p, miR-122-5p, miR-124-3p, miR-125b-5p, and miR-148a-3p, miR-21-5p, miR-23a-3p, miR-24-3p, and miR-93-5p were assayed by RT-qPCR. The results showed that circulating miR-100-5p, miR-122-5p, miR-124-3p, miR-148a-3p, miR-21-5p, miR-23a-3p, miR-24-3p, and miR-93-5p were significantly upregulated in the OP women and men compared with the controls, but miR-93-5p failed to discriminate between OP and non-OP male patients. Furthermore, miR-125b-5p expression was gender-related. In the OP bone samples, miR-100-5p, miR-125b-5p, miR-21-5p, miR-24-3p, and miR-93-5p were significantly upregulated in the OP patients compared with the controls and correlated with BMD. In particular, miR-21-5p expression values discriminated between osteopenia and OP. miR-100-5p, miR-125b-5p, miR-21-5p, miR-23a-3p, miR-24-3p, and miR-93-5p were upregulated in OP osteoblasts, while miR-100-5p, miR-122-5p, miR-124-3p, miR-125b-5p, miR-148a-3p, miR-21-5p, and miR-93-5p were upregulated in OP osteoclasts. Among these miRNAs, miR-122-5p was previously identified as being upregulated in serum samples from OP patients with bone fracture (Panach et al., 2015). The role of the other miRNAs and their potential target genes have been described above. These results identify miRNAs with high potential as biomarkers for OP, as well as targets for OP therapeutic treatment (Kelch et al., 2017). Recent studies have investigated whether single or combined miRNAs discriminate bone fractures in conditions associated with bone fragility. Interestingly, the nineteen serum miRNAs found altered in postmenopausal women by Kocijan et al. (2016), as previously described, were found altered also in serum samples from trauma fractures in idiopathic OP (premenopausal women, n = 10, and men, n = 16) compared to their controls (n = 28, 12 premenopausal women and 16 men) without bone fracture. Also in these cases, ROC analysis revealed that miR-140-5p, miR-152-3p, miR-19a-3p, miR-19b-3p, miR-30e-5p, miR-324-3p, miR-335-5p, and miR-550a-3p had a higher discriminating power between bone fracture and controls (AUC> 0.9) than BMD or bone turnover markers. Mandourah et al. (2018) recruited 139 subjects and divided them into 5 groups: healthy controls, osteopenic subjects with or without bone fractures, and OP patients with or without bone fractures. Fifteen of the 370 miRNAs screened in the pooled sera were differently regulated in the females with OP and the healthy females, and twenty-five were up or downregulated in the OP females compared with the osteopenic females. Following RT-qPCR validation, miR-122-5p and miR-4516 levels differed between the healthy subjects and the osteopenic/OP patients. Moreover, serum miR-122-5p and miR-4516 levels were lower in the OP patients than the healthy controls and osteopenic patients. miR-4516 was also found to be downregulated in the OP patients with bone fracture and associated with BMD. ROC analysis revealed that only miR-4516 had an acceptable diagnostic value for OP: AUC 0.727, 71% sensitivity, and 62% specificity. Furthermore, the diagnostic value of these two miRNAs increased when combined (AUC 0.752). Overall, these findings indicate that miR-122-5p and miR-4516 downregulation in patient samples may be associated with OP progression. However, miR-122-5p has been found upregulated in the sera of OP patients with hip fracture (Panach et al., 2015).

In order to discriminate between type 2 diabetes (T2DM)- and OP-associated bone fracture, serum levels of 375 miRNAs were evaluated using a low-density qPCR array. Forty-eight miRNAs were differentially expressed between T2DM patients with bone fracture and healthy controls, and 23 miRNAs differentially expressed between OP with bone fracture and healthy controls. Eighteen of these showed the same regulation pattern in the T2DM and the OP patients. Considering the top ten ranking miRNAs (i.e., four-miRNA model signatures with AUC values >0.9 for identifying the T2DM or OP fragility fracture groups), the most abundant miRNAs were miR-382-3p, miR-550a-5p, and miR-96-5p for the T2DM group and miR-188-3p, miR-382-3p, miR-942 for the OP group. miR-382-3p was downregulated in both groups with bone fracture compared with the controls; miR-550a-5p and miR-96-5p were significantly upregulated in the T2DM patients with bone fractures, while miR-188-3p and miR-942 were downregulated, although without reaching statistical significance, in OP bone fractures compared with the controls: these last two miRNAs are associated with bone metabolism (Heilmeier et al., 2016). miR-188 is recognized as a main modulator of the BM-MSCs age-associated osteogenesis-to-adipogenesis shift by targeting histone deacetylase 9 (HDAC9) and the RPTOR-independent companion of mTOR complex 2 (RICTOR). In particular, miR-188 suppression induces osteoblast differentiation and bone formation (Li et al., 2015a). By targeting the heparin-binding EGF-like growth factor (HB-EGF), miR-96 is able to promote osteoblast differentiation (Yang et al., 2014). Analyzing the *in vitro* effects of miR-188-3p, miR-382-3p, and miR-550a-5p on cell proliferation, osteogenesis, and adipogenesis, the authors demonstrated that miR-382-3p and miR-550a-5p enhance and inhibit, respectively, osteogenic differentiation and both affect adipogenesis, whereas miR-188-3p does not impair it. Thus, miR-382-3p and miR-550a-5p have been identified as potential circulating biomarkers for T2DM-associated bone disease, and miR-188-3p and miR-382-2p for bone fractures in OP (Heilmeier et al., 2016).

**Table 3** presents information about circulating miRNAs associated with other types of OP and related fracture risk.

**Table 3:** miRNAs associated with other types of OP and related fracture risk.

Study	Study design	Biomarker source	Sample handling	Quantification platform	Evaluated miRNA	Normalization strategy	Validated miRNA biomarker	Potential target gene	AUC-Sensitivity(%)-Specificity(%)	Limits
(Chen et al., 2014b)	4 Chinese OP patients (3 women and 1 man, age 76-88 years) and 5 Chinese subjects with normal BMD (2 women and 3 men, age 19-44 years)	BM-MCSs	Bone marrow aspirated from iliac crest and used for BM-MCSs isolation	SYBR Green RT-qPCR	miR-125b	snRNU6	↑miR-125b in OP group vs. non-OP group	OSX (Identified using TargetScan and PicTar database, and validated by *in vitro* experiments) and RUNX2 for miR-125b	/	Small sample size; no information about the stem-loop arm of miRNA origin; no ROC analysis
(Weilner et al., 2016)	14 men (mean age ∼53 years) with idiopathic osteoporosis and 11 age-matched HC	Cell-free plasma and plasma microvesicles	Filtration and differential centrifugation methodologies for microvescicle purification	TaqMan RT-qPCR	miR-31	snRNU6	↑miR-31 in OP group vs. HC	FZD3 (validated by *in vitro* experiments for miR-31)	/	Small sample size; no information about the stem-loop arm of miRNA origin; study mainly focused on miRNA evaluation by *in vitro* studies; no ROC analysis
(Wang et al., 2013c)	40 Chinese patients with fracture (age 60-90 years) and 9 Chinese HC (control group)	Bone specimens	Femurs collected during surgery	RT-PCR	Not specified	Not specified in this paper	↑miR-214a in older individuals	ATF4 (identified for miR-214a using miRBase and validated by *in vitro-in vivo* experiments)	/	Small sample size of the HC group; confusing information about the comparisons done among groups; evaluated miRNAs and data normalization not explained in this paper; no information about the stem-loop arm of miRNA origin; no ROC analysis
(Kelch et al., 2017)	28 patients with hip fracture: 7 men + 7 women with OP and 7 men + 7 women with AO (control group)	Cell-free serum and bone tissue	Blood collected 2 h post-fracture (OP) or pre-operation (non-OP) into S-Monovette polypropylene tubes, placed for 30 min at RT upright, centrifuged for 10 min at 1900g, serum stored at -80°CFemoral head samples collected during surgery (within 8 h after fracture in OP group). Cylindrical bone samples obtained from middle of each femoral head, cut into small pieces with Luer forceps, rinsed with D-PBS, collected in TRI-Reagent, snap frozen in liquid nitrogen, and mechanically ground. The bone powder collected with TRI-Reagent and stored at -80°C	miScript SYBR Green RT-qPCR	miR-21-5p, miR-23a-3p, miR-24-3p, miR-93-5p, miR-100-5p, miR-122-5p, miR-124-3p, miR-125b-5p, and miR-148a-3p	SNORD96a	↑ miR-21-5p, miR-23a-3p, miR-24-3p, miR-93-5p, miR-100-5p, miR-122-5p, miR-124-3p, and miR-148a-3p in OP serum vs. control↑ miR-21-5p, miR-24-3p, miR-93-5p, miR-100-5p and miR-125b-5p in OP tissues vs. control↑ miR-21-5p, miR-23a-3p, miR-24-3p, miR-93-5p, miR-100-5p, and miR-125b-5p in OP osteoblasts vs. control↑ miR-21-5p, miR-93-5p, miR-100-5p, miR-122-5p, miR-124-3p, miR-125b-5p, and miR-148a-3p in OP osteoclasts vs. control	/	/	no evaluation of the target genes; no ROC analysis
(Kocijan et al., 2016)	Patients with idiopathic (16 men and 10 premenopausal women); HC without low-trauma fracture (16 men and 12 premenopausal women).	Cell-free serum	Fasting blood samples immediately centrifuged and serum stored a -80°C	SYBR Green RT-qPCR	187 miRNAs tested	Global mean	↑ miR-152-3p, miR-335-5p, miR-320a and↓ let-7b-5p, miR-7-5p, miR-16-5p, miR-19a-3p, miR-19b-3p, miR-29b-3p, miR-30e-5p, miR-93-5p, miR-140-5p, miR-215-5p, miR-186-5p, miR-324-3p, miR-365a-3p, miR-378a-5p, miR-532-5p, and miR-550a-3p in fractured groups vs. their control groups	/	0.962 (miR-152-3p), 0.959 (miR-30e-5p), 0.950 (miR-324-3p), 0.947(miR-140-5p), 0.944 (miR-19b-3p), 0.939 (miR-335-5p), 0.929 (miR-19a-3p), 0.909 (miR-550a-3p), 0.898 (miR-186-5p), 0.898 (miR-532-5p), 0.872 (miR-378a-5p), 0.870 (miR-320a), 0.879 (miR-93-5p), 0.857 (miR-16-5p), 0.853 (miR-215-5p), 0.852 (let-7b-5p), 0.824 (miR-7-5p), 0.838 (miR-29b-3p), and 0.809 (miR-365a-3p) for fracture groups vs. control groups	No evaluation of the target genes; arbitrary choice of the screened miRNAs
(Mandourah et al., 2018)	12 (1 male/11 females) non-OP controls, 61 (9 males/52 females) osteopenia without fracture, 15 (2 males/13 females) osteopenia with fracture, 33 (6 males/27 females) OP without fracture, and 18 (2 males/16 females) OP with fracture	Cell-free serum and plasma	Serum/plasma samples obtained by centrifuging at 2500g and RT for 30 min. Supernatants further centrifuged at 14000g and 4°C for 30 min. Samples stored at -80°C	Screening: Human Serum and Plasma miRNA PCR arrays Validation: miScript SYBR Green RT-qPCR	Screening: 370 miRNAs tested Validation: 40 miRNAs tested	SNORD96A and RNU6-6P	↓ miR-122-5p and miR-4516 in OP vs. non-OP and osteopenia patients	BMP2K, FSHB, IGF1R, VDR, SPARC, TSC22D3 and RUNX2 (miR-122-5p and miR-4516); ANKH, ALPL, CNR2, CD44, LRP6, and ESR1 (miR-122-5p); AR and CNR1 (miR-4516) (identified using miRWalk2.0 database but not validated in the study)	0.727-71-62 (miR-4516) and 0.752 (miR-122-5p+miR-4516) for OP	Small sample size; confusing information about the comparisons done among groups; no validation of the identified target genes
(Heilmeier et al., 2016)	80 PM women; two study arms with two groups each:T2DM arm composed of T2DM women with (n = 20) and without (n = 20) fragility fractures since T2DM onsetOP arm composed of healthy non-T2DM PM women with OP fragility fracture (n = 20), and control group of fracture-free PM women (n = 20).	Cell-free serum	Fasting blood placed for 40 min upright and centrifuged for 15 min at 2000g.	SYBR Green Low-density qPCR platform	375 miRNAs tested	Cq values computed using second derivative maximum method provided with LC480 II software.	Most abundant miRNAs among the top 10 four-miRNAs models:↓ miR-382-3p in T2DM and OP with fragility fracture vs. respective controls↑ miR-550a-5p and miR-96-5p in T2DM fragility fracture group vs. controls↓ miR-188-3p and miR-942 in OP fracture group vs. controls	/	10 candidate four-miRNA models displayed AUC values (0.922 -0.965) for identifying fracture status in T2DM.10 candidate four-miRNA models displayed AUC values (0.972 -0.991) for identifying fracture status in OP group.	No evaluation of the target genes; arbitrary choice of the screened miRNAs

ALPL, alkaline phosphatase; ANKH, ANKH inorganic pyrophosphate transport regulator; AR, androgen receptor; ATF4, activating transcription factor 4; BMD, bone mineral density; BM-MCSs, bone marrow mesenchymal stem cells; BMP2K, BMP2 inducible kinase; CNR2, cannabinoid receptor 2; ESR1, estrogen receptor 1; FSHB, follicle stimulating hormone subunit beta;FZD3, frizzled-3; HC, healthy controls; IGF1R, insulin-like growth factor 1 receptor;OA, osteoarthritis; OP, osteoporosis; OSX, osterix; RT-qPCR, real-time quantitative polymerase chain reaction; RUNX2, runt-related transcription factor 2; RUNX2, runt-related transcription factor 2; SPARC, secreted protein acidic and cysteine rich; T2DM, type 2 diabetes mellitus; TSC22D3, TSC22 domain family member 3; VDR, vitamin D receptor; WIF1, WNT inhibitory factor 1; WISP1, WNT1-inducible-signaling pathway protein 1.

### Conclusions

The growing body of evidence for the fundamental modulatory role exerted by miRNAs in biological functions, along with aberrant expression in disease onset, underline their potential as biomarkers for the onset and progression of disease. Based on current evidence, age-related bone diseases, especially in OP and OP fractures, may be correlated with altered levels of circulating and tissue miRNA. In addition, the essential regulatory role exerted by miRNAs in bone homeostasis, as revealed by *in vitro* and *in vivo* studies, underscores their huge potential as biomarkers for diagnosis, prognosis, and personalized treatment of age-associated bone-related disease. Unfortunately, clinical studies for identifying circulating miRNAs as markers for bone diseases have employed various different experimental protocols, making it difficult to compare the results obtained from different labs and even from the same lab in some cases. Furthermore, the great majority of the published studies, here reviewed, are featured by limited (and sometimes statistically unjustifiably too limited) sample sizes. For these reasons, more effort must be spent in standardizing the pre-analytical, analytical, and post-analytical stage of miRNAs discovery and validation to obtain valuable biomarkers for clinical practice and to improve the significance by validating, at least the most promising biomarkers, on wide and real life-adherent populations.

## Author Contributions

MB: Drafting the work, final approval. GB: Conception of the work, critical revision, final approval. GL: Conception of the work, drafting the work, critical revision, final approval.

## Funding

This study was funded by the Italian Ministry of Health (Ricerca Corrente).

## Conflict of Interest

The authors declare that the research was conducted in the absence of any commercial or financial relationships that could be construed as a potential conflict of interest.
